# Supplementary material for: Criteria for evaluating molecular markers: Comprehensive quality metrics to improve marker-assisted selection
Source: PLoS One. 2019 Jan 15;14(1):e0210529. doi: 10.1371/journal.pone.0210529 (PMC6333336; doi:10.1371/journal.pone.0210529)
Supplement: S1 Table — (PDF) [file pone.0210529.s001.pdf]

Supplemental Table 1: List of indel and SSR markers assessed for technical performance

Marker	Chromosome	Position	Marker type
1_21705593	1	21705593	Indel
1_27801952	1	27801952	Indel
1_32254725	1	32254725	Indel
1_34198604	1	34198604	Indel
1_36293501	1	36293501	Indel
1_37231928	1	37231928	Indel
1_37588170	1	37588170	Indel
1_37698557	1	37698557	Indel
1_37724349	1	37724349	Indel
1_37796891	1	37796891	Indel
1_37903562	1	37903562	Indel
Os01g0874100	1	37918160	Indel
1_39501901	1	39501901	Indel
MSU6_2_973068_[Indel]	2	973068	Indel
MSU6_2_3949702_[Indel]	2	3949702	Indel
MSU6_2_5241905_[Indel]	2	5241905	Indel
2_7499561	2	7499561	Indel
2_8594377	2	8594377	Indel
2_9003534	2	9003534	Indel
2_10315707	2	10315707	Indel
2_10865762	2	10865762	Indel
2_11120246	2	11120246	Indel
2_11265804	2	11265804	Indel
2_13611036	2	13611036	Indel
3_179286	3	179286	Indel
3_190373	3	190373	Indel
3_1850409	3	1850409	Indel
3_2163413	3	2163413	Indel
3_3775875	3	3775875	Indel
3_4519585	3	4519585	Indel
3_4800740	3	4800740	Indel
3_7018296	3	7018296	Indel
3_7035021	3	7035021	Indel
3_7231633	3	7231633	Indel
3_8062383	3	8062383	Indel
3_27422379	3	27422379	Indel
3_28739422	3	28739422	Indel
LOC_Os03g50470	3	28798244	Indel
3_28965396	3	28965396	Indel
3_28975124	3	28975124	Indel
3_29117723	3	29117723	Indel
3_29604053	3	29604053	Indel
3_29660435	3	29660435	Indel
3_33106036	3	33106036	Indel
3_33118992	3	33118992	Indel
3_34931082	3	34931082	Indel
3_35574371	3	35574371	Indel
MSU6_4_196102_[Indel]	4	196102	Indel

Supplemental Table 1: List of indel and SSR markers assessed for technical performance

Marker	Chromosome	Position	Marker type
MSU6_4_1068894_[Indel]	4	1068894	Indel
MSU6_4_1329641_[Indel]	4	1329641	Indel
MSU6_4_1977984_[indel]	4	1977984	Indel
MSU6_4_2281606_[Indel]	4	2281606	Indel
MSU6_4_17302271_[Indel]	4	17302271	Indel
MSU6_4_17414046_[Indel]	4	17414046	Indel
MSU6_4_17536240_[Indel]	4	17536240	Indel
MSU6_4_17987548_[Indel]	4	17987548	Indel
MSU6_4_18081825_[Indel]	4	18081825	Indel
MSU6_4_18349442_[Indel]	4	18349442	Indel
MSU6_4_19012894_[Indel]	4	19012894	Indel
MSU7_5_438902_[0/-35]	5	438902	Indel
MSU7_6_1767284_[Indel]	6	1767284	Indel
MSU7_6_6754433_[Indel]	6	6754433	Indel
7_218555	7	218555	Indel
7_503640	7	503640	Indel
7_821345	7	821345	Indel
7_1288962	7	1288962	Indel
7_3500120	7	3500120	Indel
7_4854494	7	4854494	Indel
7_6062026 bHLH	7	6062026	Indel
7_8052183 MYB	7	8052183	Indel
7_8152295	7	8152295	Indel
Os08g0424500 BADH2	8	20380144	Indel
11_2456241	11	2456241	Indel
MSU7_11_3765242_[Indel]	11	3765242	Indel
MSU7_11_3914993_[Indel]	11	3914993	Indel
MSU7_11_4116877_[Indel]	11	4116877	Indel
MSU7_11_4301013_[Indel]	11	4301013	Indel
11_4328892	11	4328892	Indel
MSU7_11_4439870_[Indel]	11	4439870	Indel
MSU7_11_5173655_[Indel]	11	5173655	Indel
12_3024705	12	3024705	Indel
12_7280706	12	7280706	Indel
12_9799992	12	9799992	Indel
12_10965030	12	10965030	Indel
12_14112244	12	14112244	Indel
12_22063196	12	22063196	Indel
RM10115	1	2154332	SSR
RM490	1	6676195	SSR
RM3412b	1	11566961	SSR
RM493	1	12264091	SSR
RM140	1	12284725	SSR
RM10864	1	14236113	SSR
RM7075	1	15101795	SSR
RM3482	1	39713330	SSR
RM14	1	41362794	SSR
RM12208	1	41935041	SSR

Supplemental Table 1: List of indel and SSR markers assessed for technical performance

Marker	Chromosome	Position	Marker type
RM5626	3	24671259	SSR
RM6329	3	28608271	SSR
RM3867	3	31540070	SSR
R3M53	3	32255684	SSR
RM7076	3	33446993	SSR
RM7389	3	35948517	SSR
RM11	7	19204350	SSR
RM172	7	29561293	SSR
RM23877	9	6347247	SSR
RM17	12	27024527	SSR
