# Supplementary material for: Criteria for evaluating molecular markers: Comprehensive quality metrics to improve marker-assisted selection
Source: PLoS One. 2019 Jan 15;14(1):e0210529. doi: 10.1371/journal.pone.0210529 (PMC6333336; doi:10.1371/journal.pone.0210529)
Supplement: S2 Table — (PDF) [file pone.0210529.s002.pdf]

Supplemental Table 2: List of varieties examined for technical performance evaluation.

Variety name
Azucena
BINA dhan 10
BINA dhan 8
Boilam
BR11
BR24
BR28
BR29
BRRI dhan 55
BRRI dhan 61
Capsule
Cheriviruppu
Co39
CR dhan 405
CSR 28
CSR 36
CSR-89 IR-15
CSR-90 IR-2
Daw Hawm
Eratio
FL478
Gundang
IR28
IR29
IR42
IR5
IR56
IR64
IR72046-B-R-3
IR74
IR77298-14-1-2-10
IR8
IRRI 101
IRRI 102
IRRI 103
IRRI 104
IRRI 105
IRRI 106
IRRI 108
IRRI 109
IRRI 110
IRRI 111
IRRI 112
IRRI 113
IRRI 114
IRRI 115
IRRI 116
IRRI 117

Supplemental Table 2: List of varieties examined for technical performance evaluation.

Variety name
IRRI 118
IRRI 119
IRRI 120
IRRI 121
IRRI 122
IRRI 123
IRRI 124
IRRI 125
IRRI 126
IRRI 127
IRRI 128
IRRI 129
IRRI 130
IRRI 131
IRRI 132
IRRI 133
IRRI 134
IRRI 135
IRRI 136
IRRI 138
IRRI 139
IRRI 140
IRRI 141
IRRI 142
IRRI 143
IRRI 145
IRRI 146
IRRI 147
IRRI 148
IRRI 149
IRRI 150
IRRI 151
IRRI 152
IRRI 154
IRRI 155
IRRI 156
IRRI 157
IRRI 158
IRRI 159
IRRI 160
IRRI 161
IRRI 162
IRRI 163
IRRI 164
IRRI 165
IRRI 167
IRRI 168
IRRI 169

Supplemental Table 2: List of varieties examined for technical performance evaluation.

Variety name
IRRI 170
IRRI 171
IRRI 172
IRRI 173
IRRI 174
IRRI 175
IRRI 176
IRRI 177
IRRI 178
IRRI 179
IRRI 180
IRRI 181
IRRI 184
IRRI 185
Jumbo Jet
Kala Rata
Makassane
Moroberekan
Nona Bokra
POKKALI
Pokkali 26869
Pokkali 8948
PSB Rc82
Sambha Mahsuri-Sub1
SR 26 b
