# Supplementary material for: Criteria for evaluating molecular markers: Comprehensive quality metrics to improve marker-assisted selection
Source: PLoS One. 2019 Jan 15;14(1):e0210529. doi: 10.1371/journal.pone.0210529 (PMC6333336; doi:10.1371/journal.pone.0210529)
Supplement: S3 Table — (PDF) [file pone.0210529.s003.pdf]

Supplemental Table 3. List of varieties used in calculating biological accuracy and breeding metrics.

Name	Cultivation Status
FL478	Breeding line
IR77298-5-6-11	Breeding line
IR81896-B-B-195	Breeding line
IR81896-B-B-431	Breeding line
IR81896-B-B-74	Breeding line
IRBB60	Breeding line
IRBB62	Breeding line
IRBB7	Breeding line
Aomierte 168	Cultivar
Apo	Cultivar
ASD1	Cultivar
Avo	Cultivar
Bala	Cultivar
Banjiemang 2	Cultivar
BG300	Cultivar
BG304	Cultivar
BG90-2	Cultivar
BG94-1	Cultivar
BINA dhan 10	Cultivar
Binam	Cultivar
BR11	Cultivar
BR24	Cultivar
BR28	Cultivar
BR29	Cultivar
Co22	Cultivar
Co39	Cultivar
CR dhan 405	Cultivar
CR147-392-4	Cultivar
CSR89	Cultivar
CSR90	Cultivar
Cypress	Cultivar
Dasanbyeo	Cultivar
Dinorado	Cultivar
Drew	Cultivar
G Zhenshan 97B	Cultivar
GalsaegSsal Sharei	Cultivar
GIZA159	Cultivar
Giza178	Cultivar
Hnankar	Cultivar
IAC165	Cultivar
IAC3	Cultivar
IAC47	Cultivar
IET1444	Cultivar
IR02A127	Cultivar
IR03A568	Cultivar
IR04A115	Cultivar
IR05N412	Cultivar
IR06A150	Cultivar

Supplemental Table 3. List of varieties used in calculating biological accuracy and breeding metrics.

Name	Cultivation Status
IR06G113	Cultivar
IR06N209	Cultivar
IR07A234	Cultivar
IR07F287	Cultivar
IR07F290	Cultivar
IR08A172	Cultivar
IR08A176	Cultivar
IR08N194	Cultivar
IR09A136	Cultivar
IR09A138	Cultivar
IR09F436	Cultivar
IR09N190	Cultivar
IR09N496	Cultivar
IR09N514	Cultivar
IR09N530	Cultivar
IR09N531	Cultivar
IR09N538	Cultivar
IR09N542	Cultivar
IR10179-23-1-3	Cultivar
IR10F365	Cultivar
IR10N108	Cultivar
IR22	Cultivar
IR24	Cultivar
IR26	Cultivar
IR28	Cultivar
IR29	Cultivar
IR31917	Cultivar
IR34	Cultivar
IR42	Cultivar
IR42	Cultivar
IR45427-2B-2-2B-1-1	Cultivar
IR55419	Cultivar
IR56	Cultivar
IR58025B	Cultivar
IR58821-23-1-3-1	Cultivar
IR6	Cultivar
IR62266-42-6-2	Cultivar
IR64	Cultivar
IR64A	Cultivar
IR65600-27-1-2-2	Cultivar
IR66897B	Cultivar
IR66946-3R-178-1-1	Cultivar
IR68	Cultivar
IR68552-55-3-2	Cultivar
IR71466-75-3-B-1	Cultivar
IR73004-3-1-2-1	Cultivar
IR74	Cultivar
IR77298-14-1-2-10	Cultivar

Supplemental Table 3. List of varieties used in calculating biological accuracy and breeding metrics.

Name	Cultivation Status
IR8	Cultivar
IR8192-200-3-3-1-1	Cultivar
IRAT352	Cultivar
IRBB23	Cultivar
IRBB61	Cultivar
IRBB62	Cultivar
IRBB64	Cultivar
IRBB65	Cultivar
IRRI110	Cultivar
IRRI119	Cultivar
IRRI123	Cultivar
IRRI139	Cultivar
IRRI146	Cultivar
IRRI146	Cultivar
IRRI147	Cultivar
IRRI147	Cultivar
IRRI154	Cultivar
IRRI168	Cultivar
IRRI171	Cultivar
IRRI174	Cultivar
IRRI181	Cultivar
Jinbubyeo	Cultivar
Katy	Cultivar
Kotobuki-Mochi	Cultivar
Lemont	Cultivar
Lijian 942	Cultivar
Litchikiang	Cultivar
LTH	Cultivar
M202	Cultivar
M23	Cultivar
M401	Cultivar
Makassane	Cultivar
Manaw-Thuka	Cultivar
Matatag2	Cultivar
Milyang 30	Cultivar
Milyang 77	Cultivar
MILYANG23	Cultivar
Minghui 63	Cultivar
Monolaya	Cultivar
MS11	Cultivar
MTU1010	Cultivar
NERICA-1	Cultivar
NERICA-2	Cultivar
NERICA-2	Cultivar
NERICA-8	Cultivar
NERICA-9	Cultivar
NERICA-L-20	Cultivar
NERICA-L-27	Cultivar

Supplemental Table 3. List of varieties used in calculating biological accuracy and breeding metrics.

Name	Cultivation Status
NSIC Rc222	Cultivar
OM1706	Cultivar
OM997	Cultivar
Pankhari 203	Cultivar
PSB Rc10	Cultivar
PSB Rc18	Cultivar
PSB Rc18	Cultivar
PSB Rc50	Cultivar
PSB Rc68	Cultivar
PSB Rc80	Cultivar
PSB Rc82	Cultivar
PSB Rc86	Cultivar
PSB Rc88	Cultivar
Pusa Basmati 1	Cultivar
Sambha Mahsuri-Sub1	Cultivar
San Huang Zhan No. 2	Cultivar
SR26B	Cultivar
Sri Lanka 1	Cultivar
Swarna	Cultivar
Swarna-Sub1	Cultivar
Tadukan	Cultivar
TDK1	Cultivar
TKM9	Cultivar
TN1	Cultivar
TOG5674	Cultivar
Tongil 101	Cultivar
Tongil 78	Cultivar
Vandana	Cultivar
Way Rarem	Cultivar
4583	Landrace
36067-1	Landrace
498-2A-BR-8	Landrace
Adday Sel	Landrace
ADR52	Landrace
Angifotsy	Landrace
ARC12536	Landrace
Azucena	Landrace
Baiang 6	Landrace
Basmati 1	Landrace
Basmati 385	Landrace
Beizinuo	Landrace
Bikyat	Landrace
Bue Ga Wa	Landrace
Bueng Mong Leng Wei	Landrace
Capsule	Landrace
Daw Hawm	Landrace
Dhola Aman	Landrace
E Daw Hawm	Landrace

Supplemental Table 3. List of varieties used in calculating biological accuracy and breeding metrics.

Name	Cultivation Status
Eratio	Landrace
Fan Geng 6	Landrace
Fanhaopi	Landrace
Flipper	Landrace
Gundang	Landrace
Hasawi	Landrace
Honderawala	Landrace
JumboJet	Landrace
Kali Aus	Landrace
Kasalath	Landrace
KDML105	Landrace
Khao Hlan On	Landrace
Khao Nan	Landrace
Kharsu	Landrace
Kulon	Landrace
Lab	Landrace
Malchi	Landrace
Malogbana	Landrace
Ma-Zhan Red	Landrace
Moroberekan	Landrace
N22	Landrace
Nam Jam	Landrace
Nanhi	Landrace
NCS-599	Landrace
Nipponbare	Landrace
NonaBokra	Landrace
NX-3533	Landrace
Nyao	Landrace
Phaka Rumduol	Landrace
Pokkali	Landrace
Pokkali (8558)	Landrace
PTB-18	Landrace
Rayada	Landrace
Riz type Sorgho	Landrace
Roxani	Landrace
Sadri	Landrace
Sadri rice 1	Landrace
ShinchikuIku 103	Landrace
SML Awini	Landrace
Sossoka	Landrace
Taichung 65	Landrace
Tetep	Landrace
Tima	Landrace
Tjere Sugi	Landrace
TOG6542	Landrace
TOG7291	Landrace
Tres Meses	Landrace
Vary 5000 Fotsy	Landrace

Supplemental Table 3. List of varieties used in calculating biological accuracy and breeding metrics.

Name	Cultivation Status
Xiushui 115	Landrace
Zhongchao 123	Landrace
