# Supplementary material for: Criteria for evaluating molecular markers: Comprehensive quality metrics to improve marker-assisted selection
Source: PLoS One. 2019 Jan 15;14(1):e0210529. doi: 10.1371/journal.pone.0210529 (PMC6333336; doi:10.1371/journal.pone.0210529)
Supplement: S4 Table — (PDF) [file pone.0210529.s004.pdf]

Supplemental Table 4. List of marker positions interrogated for assessing biological accuracy and breeding metrics. All positions are relative to the MSU7 reference genome

Marker name	Chro-mosome	Position	Favourable allele	Unfavourable allele	Target QTL	Marker linkage	Specificity	Fingerprinting?	Source	Marker type
9558526	9	12036453	A	G	AG1	Foreground	Anonymous		Infinium chip	SNP
9563291	9	12154616	C	T	AG1	Foreground	Anonymous		Infinium chip	SNP
9565186	9	12219648	C	A	AG1	Foreground	Anonymous		Infinium chip	SNP
9569595	9	12326525	G	A	AG1	Foreground	Anonymous		Infinium chip	SNP
9578301	9	12545658	G	T	AG1	Foreground	Anonymous		Infinium chip	SNP
3921623	4	6835634	A	G	BPH17	Foreground	Anonymous		Infinium chip	SNP
id4002671	4	6913215	A	G	BPH17	Foreground	Anonymous		Infinium chip	SNP
3933751	4	7170237	C	T	BPH17	Foreground	Anonymous		Infinium chip	SNP
5859730	6	1272777	G	A	BPH3	Foreground	Anonymous	Fingerprinting	Infinium chip	SNP
id6000911	6	1378891	A	G	BPH3	Foreground	Anonymous	Fingerprinting	Infinium chip	SNP
5865517	6	1421311	A	C	BPH3	Foreground	Anonymous	Fingerprinting	Infinium chip	SNP
SNP-6_1500959	6	1501961	A	G	BPH3	Foreground	Anonymous	Fingerprinting	Infinium chip	SNP
5868825	6	1521855	A	C	BPH3	Foreground	Anonymous	Fingerprinting	Infinium chip	SNP
fd8	6	1768006	A	C	BPH3	Foreground	Anonymous	Fingerprinting	Infinium chip	SNP
4904201	5	3253516	G	A	Chalk5	Foreground	Anonymous		Infinium chip	SNP
4904312	5	3260320	C	A	Chalk5	Foreground	Anonymous		Infinium chip	SNP
4908650	5	3427089	A	G	Chalk5	Foreground	Anonymous		Infinium chip	SNP
4909480	5	3452362	C	T	Chalk5	Foreground	Anonymous		Infinium chip	SNP
4909734	5	3470087	C	A	Chalk5	Foreground	Anonymous		Infinium chip	SNP
4668476	4	30174083	T	C	COLD1	Foreground	Anonymous		Infinium chip	SNP
id4010220	4	30330971	G	A	COLD1	Foreground	Anonymous		Infinium chip	SNP
4672837	4	30362053	A	C	COLD1	Foreground	Anonymous		Infinium chip	SNP
id4010238	4	30393769	A	G	COLD1	Foreground	Anonymous		Infinium chip	SNP
4674556	4	30442759	T	C	COLD1	Foreground	Anonymous		Infinium chip	SNP
4678550	4	30601123	G	A	COLD1	Foreground	Anonymous		Infinium chip	SNP
4683917	4	30843348	G	A	COLD1	Foreground	Anonymous		Infinium chip	SNP
4683923	4	30843753	C	A	COLD1	Foreground	Anonymous		Infinium chip	SNP
4684914	4	30879875	T	C	COLD1	Foreground	Anonymous		Infinium chip	SNP
id4010433	4	30920978	A	G	COLD1	Foreground	Anonymous		Infinium chip	SNP
4689325	4	31052578	G	T	COLD1	Foreground	Anonymous		Infinium chip	SNP

Supplemental Table 4. List of marker positions interrogated for assessing biological accuracy and breeding metrics. All positions are relative to the MSU7 reference genome

Marker name	Chro-mosome	Position	Favourable allele	Unfavourable allele	Target QTL	Marker linkage	Specificity	Fingerprinting?	Source	Marker type
8638449	8	16394577	C	T	DEP1	Foreground	Anonymous		Infinium chip	SNP
8640428	8	16439432	A	G	DEP1	Foreground	Anonymous		Infinium chip	SNP
8640728	8	16443521	G	A	DEP1	Foreground	Anonymous		Infinium chip	SNP
ud8001065	8	16454443	A	G	DEP1	Foreground	Anonymous		Infinium chip	SNP
id8004340	8	16523573	G	T	DEP1	Foreground	Anonymous		Infinium chip	SNP
8107849	8	4101332	G	T	DTH8	Foreground	Anonymous		Infinium chip	SNP
8115231	8	4306618	A	G	DTH8	Foreground	Anonymous		Infinium chip	SNP
8115442	8	4310448	T	C	DTH8	Foreground	Anonymous		Infinium chip	SNP
8120894	8	4511882	C	T	DTH8	Foreground	Anonymous		Infinium chip	SNP
8122868	8	4603854	A	C	DTH8	Foreground	Anonymous		Infinium chip	SNP
id1024092	1	38024269	C	A	DTY1.1	Foreground	Anonymous		Infinium chip	SNP
id1024129	1	38049983	C	T	DTY1.1	Foreground	Anonymous		Infinium chip	SNP
1225497	1	38224245	C	T	DTY1.1	Foreground	Anonymous		Infinium chip	SNP
1226391	1	38258929	G	T	DTY1.1	Foreground	Anonymous		Infinium chip	SNP
1226694	1	38276174	C	T	DTY1.1	Foreground	Anonymous		Infinium chip	SNP
1230423	1	38406155	G	A	DTY1.1	Foreground	Anonymous		Infinium chip	SNP
1231498	1	38446499	G	T	DTY1.1	Foreground	Anonymous		Infinium chip	SNP
1237300	1	38652270	T	C	DTY1.1	Foreground	Anonymous		Infinium chip	SNP
1238760	1	38706387	C	T	DTY1.1	Foreground	Anonymous	Fingerprinting	Infinium chip	SNP
1240276	1	38760678	G	A	DTY1.1	Foreground	Anonymous		Infinium chip	SNP
1243398	1	38847770	C	T	DTY1.1	Foreground	Anonymous	Fingerprinting	Infinium chip	SNP
1250974	1	39076741	G	A	DTY1.1	Foreground	Anonymous		Infinium chip	SNP
12707111	12	17066946	C	T	DTY12.1	Foreground	Anonymous		Infinium chip	SNP
id12005832	12	17443323	C	T	DTY12.1	Foreground	Anonymous		Infinium chip	SNP
12731194	12	17546633	T	C	DTY12.1	Foreground	Anonymous		Infinium chip	SNP
12732307	12	17571428	C	T	DTY12.1	Foreground	Anonymous		Infinium chip	SNP
id12005912	12	17571574	G	A	DTY12.1	Foreground	Anonymous		Infinium chip	SNP
1653063	2	9570228	G	A	DTY2.1	Foreground	Anonymous		Infinium chip	SNP
1661393	2	9801099	T	G	DTY2.1	Foreground	Anonymous		Infinium chip	SNP
1667329	2	9963856	C	T	DTY2.1	Foreground	Anonymous		Infinium chip	SNP

Supplemental Table 4. List of marker positions interrogated for assessing biological accuracy and breeding metrics. All positions are relative to the MSU7 reference genome

Marker name	Chro-mosome	Position	Favourable allele	Unfavourable allele	Target QTL	Marker linkage	Specificity	Fingerprinting?	Source	Marker type
1667949	2	9975660	A	G	DTY2.1	Foreground	Anonymous	Fingerprinting	Infinium chip	SNP
1675311	2	10169097	C	T	DTY2.1	Foreground	Anonymous		Infinium chip	SNP
1678844	2	10274389	C	T	DTY2.1	Foreground	Anonymous		Infinium chip	SNP
1687403	2	10494416	C	T	DTY2.1	Foreground	Anonymous		Infinium chip	SNP
1689518	2	10555263	T	C	DTY2.1	Foreground	Anonymous		Infinium chip	SNP
1698201	2	10744597	C	T	DTY2.1	Foreground	Anonymous		Infinium chip	SNP
id2005033	2	10747159	T	C	DTY2.1	Foreground	Anonymous		Infinium chip	SNP
id2005152	2	10874369	T	G	DTY2.1	Foreground	Anonymous	Fingerprinting	Infinium chip	SNP
id2005182	2	10995452	A	G	DTY2.1	Foreground	Anonymous		Infinium chip	SNP
1711443	2	11052053	C	T	DTY2.1	Foreground	Anonymous		Infinium chip	SNP
1713544	2	11102127	G	T	DTY2.1	Foreground	Anonymous		Infinium chip	SNP
1713554	2	11102648	C	T	DTY2.1	Foreground	Anonymous		Infinium chip	SNP
1714923	2	11133163	G	A	DTY2.1	Foreground	Anonymous		Infinium chip	SNP
1715617	2	11153201	G	A	DTY2.1	Foreground	Anonymous		Infinium chip	SNP
1717430	2	11227552	C	T	DTY2.1	Foreground	Anonymous		Infinium chip	SNP
1725183	2	11437686	G	A	DTY2.1	Foreground	Anonymous		Infinium chip	SNP
1727846	2	11515882	G	A	DTY2.1	Foreground	Anonymous		Infinium chip	SNP
id2005453	2	11701123	A	G	DTY2.1	Foreground	Anonymous		Infinium chip	SNP
id2005462	2	11707238	G	A	DTY2.1	Foreground	Anonymous		Infinium chip	SNP
1739007	2	11861014	A	G	DTY2.1	Foreground	Anonymous		Infinium chip	SNP
1741033	2	11996866	T	G	DTY2.1	Foreground	Anonymous		Infinium chip	SNP
1741464	2	12035419	C	A	DTY2.1	Foreground	Anonymous		Infinium chip	SNP
id2000917	2	1064938	G	A	DTY2.2	Foreground	Anonymous		Infinium chip	SNP
id2001296	2	2013925	A	G	DTY2.2	Foreground	Anonymous		Infinium chip	SNP
id2001318	2	2077711	G	A	DTY2.2	Foreground	Anonymous		Infinium chip	SNP
id2001463	2	2408032	T	A	DTY2.2	Foreground	Anonymous		Infinium chip	SNP
id2001540	2	2787469	G	A	DTY2.2	Foreground	Anonymous		Infinium chip	SNP
id2001547	2	2793214	A	G	DTY2.2	Foreground	Anonymous		Infinium chip	SNP
id2002065	2	3839369	A	G	DTY2.2	Foreground	Anonymous		Infinium chip	SNP
id2002246	2	4165922	G	C	DTY2.2	Foreground	Anonymous	Fingerprinting	Infinium chip	SNP

Supplemental Table 4. List of marker positions interrogated for assessing biological accuracy and breeding metrics. All positions are relative to the MSU7 reference genome

Marker name	Chro-mosome	Position	Favourable allele	Unfavourable allele	Target QTL	Marker linkage	Specificity	Fingerprinting?	Source	Marker type
id2002281	2	4300257	A	C	DTY2.2	Foreground	Anonymous	Fingerprinting	Infinium chip	SNP
id2002330	2	4424424	C	T	DTY2.2	Foreground	Anonymous	Fingerprinting	Infinium chip	SNP
id2002606	2	4909682	C	T	DTY2.2	Foreground	Anonymous		Infinium chip	SNP
id2002811	2	5242068	T	C	DTY2.2	Foreground	Anonymous		Infinium chip	SNP
3408998	3	30119566	T	C	DTY3.1	Foreground	Anonymous		Infinium chip	SNP
3412475	3	30247826	G	T	DTY3.1	Foreground	Anonymous		Infinium chip	SNP
rd3001030	3	30253323	C	A	DTY3.1	Foreground	Anonymous		Infinium chip	SNP
3414133	3	30342031	T	G	DTY3.1	Foreground	Anonymous		Infinium chip	SNP
3417192	3	30457668	C	T	DTY3.1	Foreground	Anonymous		Infinium chip	SNP
3417884	3	30489032	T	C	DTY3.1	Foreground	Anonymous		Infinium chip	SNP
3419323	3	30545870	C	T	DTY3.1	Foreground	Anonymous		Infinium chip	SNP
3420460	3	30604896	C	T	DTY3.1	Foreground	Anonymous		Infinium chip	SNP
3427731	3	30926534	T	C	DTY3.1	Foreground	Anonymous		Infinium chip	SNP
3429923	3	31018646	A	C	DTY3.1	Foreground	Anonymous		Infinium chip	SNP
3430019	3	31025197	C	T	DTY3.1	Foreground	Anonymous		Infinium chip	SNP
3432450	3	31138388	G	T	DTY3.1	Foreground	Anonymous		Infinium chip	SNP
3433249	3	31190989	A	G	DTY3.1	Foreground	Anonymous		Infinium chip	SNP
3433307	3	31196096	G	A	DTY3.1	Foreground	Anonymous		Infinium chip	SNP
3433537	3	31205828	C	T	DTY3.1	Foreground	Anonymous		Infinium chip	SNP
3437076	3	31348032	T	C	DTY3.1	Foreground	Anonymous		Infinium chip	SNP
3437919	3	31373153	T	C	DTY3.1	Foreground	Anonymous		Infinium chip	SNP
c3p31393777	3	31393778	G	A	DTY3.1	Foreground	Anonymous		Infinium chip	SNP
id3000019	3	209351	C	T	DTY3.2	Foreground	Anonymous		Infinium chip	SNP
Fd9	3	1270943	G	A	DTY3.2	Foreground	Anonymous	Fingerprinting	Infinium chip	SNP
id3000913	3	1670761	G	A	DTY3.2	Foreground	Anonymous		Infinium chip	SNP
id3000946	3	1737794	C	T	DTY3.2	Foreground	Anonymous		Infinium chip	SNP
id4000001	4	59946	G	A	DTY4.1	Foreground	Anonymous		Infinium chip	SNP
id4000082	4	222177	C	A	DTY4.1	Foreground	Anonymous		Infinium chip	SNP
3581003	4	247628	G	A	DTY4.1	Foreground	Anonymous	Fingerprinting	Infinium chip	SNP
3581817	4	263009	C	T	DTY4.1	Foreground	Anonymous		Infinium chip	SNP

Supplemental Table 4. List of marker positions interrogated for assessing biological accuracy and breeding metrics. All positions are relative to the MSU7 reference genome

Marker name	Chro-mosome	Position	Favourable allele	Unfavourable allele	Target QTL	Marker linkage	Specificity	Fingerprinting?	Source	Marker type
3581839	4	264061	C	T	DTY4.1	Foreground	Anonymous	Fingerprinting	Infinium chip	SNP
3584866	4	310885	G	A	DTY4.1	Foreground	Anonymous		Infinium chip	SNP
3593929	4	539827	G	T	DTY4.1	Foreground	Anonymous		Infinium chip	SNP
3597102	4	603494	A	G	DTY4.1	Foreground	Anonymous	Fingerprinting	Infinium chip	SNP
3598944	4	648669	G	A	DTY4.1	Foreground	Anonymous		Infinium chip	SNP
3606371	4	796549	T	G	DTY4.1	Foreground	Anonymous		Infinium chip	SNP
3610354	4	910623	G	A	DTY4.1	Foreground	Anonymous		Infinium chip	SNP
id4000574	4	972749	G	T	DTY4.1	Foreground	Anonymous		Infinium chip	SNP
id4000585	4	1022225	G	A	DTY4.1	Foreground	Anonymous	Fingerprinting	Infinium chip	SNP
3619998	4	1047515	A	G	DTY4.1	Foreground	Anonymous		Infinium chip	SNP
3623485	4	1136986	C	T	DTY4.1	Foreground	Anonymous	Fingerprinting	Infinium chip	SNP
3624428	4	1158223	G	A	DTY4.1	Foreground	Anonymous		Infinium chip	SNP
3625825	4	1202031	C	A	DTY4.1	Foreground	Anonymous		Infinium chip	SNP
3629184	4	1283968	C	T	DTY4.1	Foreground	Anonymous		Infinium chip	SNP
3639669	4	1540529	C	A	DTY4.1	Foreground	Anonymous		Infinium chip	SNP
3647009	4	1713004	A	G	DTY4.1	Foreground	Anonymous		Infinium chip	SNP
3647133	4	1714897	A	G	DTY4.1	Foreground	Anonymous		Infinium chip	SNP
3647336	4	1718310	T	C	DTY4.1	Foreground	Anonymous		Infinium chip	SNP
3647403	4	1719865	C	T	DTY4.1	Foreground	Anonymous	Fingerprinting	Infinium chip	SNP
3655924	4	1884399	C	T	DTY4.1	Foreground	Anonymous		Infinium chip	SNP
3659698	4	1961581	C	T	DTY4.1	Foreground	Anonymous		Infinium chip	SNP
wd4000229	4	2032639	C	T	DTY4.1	Foreground	Anonymous		Infinium chip	SNP
3672849	4	2238213	G	A	DTY4.1	Foreground	Anonymous		Infinium chip	SNP
3674743	4	2284046	G	A	DTY4.1	Foreground	Anonymous		Infinium chip	SNP
3682819	4	2394889	T	G	DTY4.1	Foreground	Anonymous	Fingerprinting	Infinium chip	SNP
3684750	4	2427802	A	C	DTY4.1	Foreground	Anonymous		Infinium chip	SNP
id4001090	4	2456175	G	A	DTY4.1	Foreground	Anonymous		Infinium chip	SNP
id4001113	4	2484571	G	A	DTY4.1	Foreground	Anonymous	Fingerprinting	Infinium chip	SNP
3710428	4	2855673	G	A	DTY4.1	Foreground	Anonymous		Infinium chip	SNP
3745693	4	3411505	a	c	DTY4.1	Foreground	Anonymous		Infinium chip	SNP

Supplemental Table 4. List of marker positions interrogated for assessing biological accuracy and breeding metrics. All positions are relative to the MSU7 reference genome

Marker name	Chro-mosome	Position	Favourable allele	Unfavourable allele	Target QTL	Marker linkage	Specificity	Fingerprinting?	Source	Marker type
8822667	8	20213731	A	G	frg-1	Foreground	Anonymous		Infinium chip	SNP
id8005525	8	20391635	G	A	frg-1	Foreground	Anonymous		Infinium chip	SNP
8829560	8	20413803	G	A	frg-1	Foreground	Anonymous		Infinium chip	SNP
163177	1	5168030	C	T	Gn1a	Foreground	Anonymous		Infinium chip	SNP
rd1000052	1	5210520	C	T	Gn1a	Foreground	Anonymous		Infinium chip	SNP
167505	1	5324684	G	A	Gn1a	Foreground	Anonymous		Infinium chip	SNP
id1004256	1	5333883	A	G	Gn1a	Foreground	Anonymous		Infinium chip	SNP
173198	1	5510350	G	T	Gn1a	Foreground	Anonymous		Infinium chip	SNP
176306	1	5608892	C	T	Gn1a	Foreground	Anonymous		Infinium chip	SNP
2932640	3	16688962	A	C	GS3	Foreground	Anonymous		Infinium chip	SNP
2934100	3	16731513	T	C	GS3	Foreground	Anonymous		Infinium chip	SNP
wd3000590	3	16733872	T	G	GS3	Foreground	Anonymous		Infinium chip	SNP
2941650	3	16889939	G	A	GS3	Foreground	Anonymous		Infinium chip	SNP
6008741	6	6508525	G	A	GT - SSIIa	Foreground	Anonymous		Infinium chip	SNP
6013900	6	6649871	T	G	GT - SSIIa	Foreground	Anonymous		Infinium chip	SNP
6015324	6	6691213	T	G	GT - SSIIa	Foreground	Anonymous		Infinium chip	SNP
6022073	6	6891960	A	C	GT - SSIIa	Foreground	Anonymous		Infinium chip	SNP
4967315	5	5256714	C	T	GW5/SW5	Foreground	Anonymous		Infinium chip	SNP
id5002699	5	5294341	T	C	GW5/SW5	Foreground	Anonymous		Infinium chip	SNP
4968944	5	5310401	G	A	GW5/SW5	Foreground	Anonymous		Infinium chip	SNP
4970110	5	5338205	T	C	GW5/SW5	Foreground	Anonymous		Infinium chip	SNP
4979148	5	5557745	G	T	GW5/SW5	Foreground	Anonymous		Infinium chip	SNP
4979702	5	5588965	T	G	GW5/SW5	Foreground	Anonymous		Infinium chip	SNP
id3000589	3	996213	T	G	Hd9	Foreground	Anonymous		Infinium chip	SNP
2506194	3	1155137	G	T	Hd9	Foreground	Anonymous		Infinium chip	SNP
4298233	4	17518575	G	A	HTSF4.1	Foreground	Anonymous		Infinium chip	SNP
4299651	4	17551912	G	A	HTSF4.1	Foreground	Anonymous		Infinium chip	SNP
4303651	4	17640628	C	T	HTSF4.1	Foreground	Anonymous		Infinium chip	SNP
4304635	4	17656765	C	T	HTSF4.1	Foreground	Anonymous		Infinium chip	SNP
4312512	4	17870318	C	T	HTSF4.1	Foreground	Anonymous	Fingerprinting	Infinium chip	SNP

Supplemental Table 4. List of marker positions interrogated for assessing biological accuracy and breeding metrics. All positions are relative to the MSU7 reference genome

Marker name	Chro-mosome	Position	Favourable allele	Unfavourable allele	Target QTL	Marker linkage	Specificity	Fingerprinting?	Source	Marker type
4313620	4	17891640	T	C	HTSF4.1	Foreground	Anonymous		Infinium chip	SNP
4314701	4	17911519	A	G	HTSF4.1	Foreground	Anonymous	Fingerprinting	Infinium chip	SNP
rd4001270	4	18212911	C	T	HTSF4.1	Foreground	Anonymous		Infinium chip	SNP
4329392	4	18253694	A	G	HTSF4.1	Foreground	Anonymous		Infinium chip	SNP
4331163	4	18320014	A	C	HTSF4.1	Foreground	Anonymous		Infinium chip	SNP
4333768	4	18408626	A	C	HTSF4.1	Foreground	Anonymous		Infinium chip	SNP
ud4001319	4	18706317	G	A	HTSF4.1	Foreground	Anonymous		Infinium chip	SNP
4344439	4	18738202	C	T	HTSF4.1	Foreground	Anonymous		Infinium chip	SNP
4349423	4	18898900	A	C	HTSF4.1	Foreground	Anonymous		Infinium chip	SNP
id2010564	2	24693023	A	G	LTG1	Foreground	Anonymous		Infinium chip	SNP
2178948	2	24712704	G	A	LTG1	Foreground	Anonymous		Infinium chip	SNP
2181296	2	24772301	A	C	LTG1	Foreground	Anonymous		Infinium chip	SNP
2181688	2	24780754	A	C	LTG1	Foreground	Anonymous		Infinium chip	SNP
4693866	4	31190068	G	A	NAL1	Foreground	Anonymous		Infinium chip	SNP
4698567	4	31350359	T	C	NAL1	Foreground	Anonymous		Infinium chip	SNP
4701155	4	31419137	C	T	NAL1	Foreground	Anonymous		Infinium chip	SNP
8983572	8	25757847	T	G	PGWC8-2	Foreground	Anonymous		Infinium chip	SNP
8984680	8	25795906	G	A	PGWC8-2	Foreground	Anonymous		Infinium chip	SNP
8989835	8	26020138	T	C	PGWC8-2	Foreground	Anonymous		Infinium chip	SNP
8990744	8	26038950	G	A	PGWC8-2	Foreground	Anonymous		Infinium chip	SNP
8991729	8	26074700	A	G	PGWC8-2	Foreground	Anonymous		Infinium chip	SNP
id1020384	1	32898485	G	A	Pi35(t)	Foreground	Anonymous		Infinium chip	SNP
1085563	1	33145570	T	C	Pi35(t)	Foreground	Anonymous		Infinium chip	SNP
1086147	1	33164035	G	A	Pi35(t)	Foreground	Anonymous		Infinium chip	SNP
1088950	1	33268652	A	C	Pi35(t)	Foreground	Anonymous		Infinium chip	SNP
1090447	1	33321905	G	A	Pi35(t)	Foreground	Anonymous		Infinium chip	SNP
id11009687	11	24983484	A	G	Pi54	Foreground	Anonymous		Infinium chip	SNP
11837165	11	25132894	G	A	Pi54	Foreground	Anonymous		Infinium chip	SNP
11837210	11	25135188	G	A	Pi54	Foreground	Anonymous		Infinium chip	SNP
11840487	11	25212665	T	G	Pi54	Foreground	Anonymous		Infinium chip	SNP

Supplemental Table 4. List of marker positions interrogated for assessing biological accuracy and breeding metrics. All positions are relative to the MSU7 reference genome

Marker name	Chro-mosome	Position	Favourable allele	Unfavourable allele	Target QTL	Marker linkage	Specificity	Fingerprinting?	Source	Marker type
11846033	11	25359525	A	G	Pi54	Foreground	Anonymous		Infinium chip	SNP
11846268	11	25368735	A	G	Pi54	Foreground	Anonymous		Infinium chip	SNP
11846273	11	25369009	G	A	Pi54	Foreground	Anonymous		Infinium chip	SNP
id11009984	11	25432067	G	A	Pi54	Foreground	Anonymous		Infinium chip	SNP
11853141	11	25531988	A	G	Pi54	Foreground	Anonymous		Infinium chip	SNP
6137646	6	10049864	C	T	Pi9	Foreground	Anonymous		Infinium chip	SNP
6147112	6	10199497	C	T	Pi9	Foreground	Anonymous		Infinium chip	SNP
6153803	6	10395448	G	T	Pi9	Foreground	Anonymous		Infinium chip	SNP
6154651	6	10418594	A	G	Pi9	Foreground	Anonymous		Infinium chip	SNP
6155433	6	10438538	G	A	Pi9	Foreground	Anonymous		Infinium chip	SNP
6167468	6	10677685	A	G	Pi9	Foreground	Anonymous		Infinium chip	SNP
12365900	12	9762923	T	C	Pita2	Foreground	Anonymous		Infinium chip	SNP
12365932	12	9763462	A	G	Pita2	Foreground	Anonymous		Infinium chip	SNP
12365977	12	9764377	T	C	Pita2	Foreground	Anonymous		Infinium chip	SNP
12379451	12	10051752	G	T	Pita2	Foreground	Anonymous		Infinium chip	SNP
12384668	12	10148623	G	A	Pita2	Foreground	Anonymous		Infinium chip	SNP
12389497	12	10228185	A	G	Pita2	Foreground	Anonymous		Infinium chip	SNP
12396903	12	10395725	A	G	Pita2	Foreground	Anonymous		Infinium chip	SNP
id12004047	12	10469146	A	G	Pita2	Foreground	Anonymous		Infinium chip	SNP
12401704	12	10509878	A	G	Pita2	Foreground	Anonymous		Infinium chip	SNP
12409876	12	10671246	T	C	Pita2	Foreground	Anonymous		Infinium chip	SNP
12413246	12	10740779	A	G	Pita2	Foreground	Anonymous		Infinium chip	SNP
12416805	12	10810714	C	T	Pita2	Foreground	Anonymous		Infinium chip	SNP
id1023347	1	37030733	A	G	qNa1L	Background-recomb	Anonymous		Infinium chip	SNP
1202195	1	37230818	T	G	qNa1L	Background-recomb	Anonymous		Infinium chip	SNP
1203155	1	37270661	A	C	qNa1L	Background-recomb	Anonymous		Infinium chip	SNP
1204367	1	37332228	T	C	qNa1L	Background-recomb	Anonymous		Infinium chip	SNP
1207615	1	37452003	T	C	qNa1L	Background-recomb	Anonymous		Infinium chip	SNP
1211309	1	37624054	T	C	qNa1L	Background-recomb	Anonymous		Infinium chip	SNP
1212226	1	37676794	G	A	qNa1L	Background-recomb	Anonymous		Infinium chip	SNP

Supplemental Table 4. List of marker positions interrogated for assessing biological accuracy and breeding metrics. All positions are relative to the MSU7 reference genome

Marker name	Chro-mosome	Position	Favourable allele	Unfavourable allele	Target QTL	Marker linkage	Specificity	Fingerprinting?	Source	Marker type
1212517	1	37692801	C	T	qNa1L	Background-recomb	Anonymous		Infinium chip	SNP
id1023824	1	37725124	T	C	qNa1L	Background-recomb	Anonymous		Infinium chip	SNP
1213474	1	37744697	G	A	qNa1L	Background-recomb	Anonymous		Infinium chip	SNP
1216964	1	37877061	C	T	qNa1L	Foreground	Anonymous		Infinium chip	SNP
1217224	1	37885159	T	C	qNa1L	Foreground	Anonymous		Infinium chip	SNP
1251387	1	39094696	C	T	qNa1L	Foreground	Anonymous		Infinium chip	SNP
1256276	1	39244791	G	A	qNa1L	Foreground	Anonymous		Infinium chip	SNP
1257104	1	39282883	G	A	qNa1L	Foreground	Anonymous		Infinium chip	SNP
1259171	1	39342234	G	A	qNa1L	Foreground	Anonymous		Infinium chip	SNP
1259861	1	39362559	G	A	qNa1L	Foreground	Anonymous		Infinium chip	SNP
id1024973	1	39369209	G	A	qNa1L	Foreground	Anonymous		Infinium chip	SNP
1261182	1	39408253	T	C	qNa1L	Foreground	Anonymous		Infinium chip	SNP
1261519	1	39420824	A	G	qNa1L	Foreground	Anonymous		Infinium chip	SNP
1264940	1	39541771	A	C	qNa1L	Foreground	Anonymous		Infinium chip	SNP
1265337	1	39556216	A	G	qNa1L	Foreground	Anonymous		Infinium chip	SNP
1271624	1	39791791	A	G	qNa1L	Foreground	Anonymous		Infinium chip	SNP
id1025292	1	39799820	A	C	qNa1L	Foreground	Anonymous		Infinium chip	SNP
1272679	1	39833556	C	A	qNa1L	Foreground	Anonymous		Infinium chip	SNP
id1025367	1	39878951	G	A	qNa1L	Foreground	Anonymous		Infinium chip	SNP
1275374	1	39971552	A	C	qNa1L	Foreground	Anonymous		Infinium chip	SNP
1275423	1	39976095	C	T	qNa1L	Foreground	Anonymous		Infinium chip	SNP
id1025455	1	39993949	G	A	qNa1L	Foreground	Anonymous		Infinium chip	SNP
1277001	1	40032941	G	T	qNa1L	Foreground	Anonymous		Infinium chip	SNP
1280193	1	40154802	G	A	qNa1L	Foreground	Anonymous		Infinium chip	SNP
1285866	1	40398232	T	G	qNa1L	Foreground	Anonymous		Infinium chip	SNP
1286531	1	40425312	C	T	qNa1L	Foreground	Anonymous		Infinium chip	SNP
c1p40455715	1	40455716	G	A	qNa1L	Foreground	Anonymous		Infinium chip	SNP
1290367	1	40551798	G	A	qNa1L	Foreground	Anonymous		Infinium chip	SNP
1295108	1	40729888	C	T	qNa1L	Foreground	Anonymous		Infinium chip	SNP
1303708	1	40997165	T	C	qNa1L	Foreground	Anonymous		Infinium chip	SNP

Supplemental Table 4. List of marker positions interrogated for assessing biological accuracy and breeding metrics. All positions are relative to the MSU7 reference genome

Marker name	Chro-mosome	Position	Favourable allele	Unfavourable allele	Target QTL	Marker linkage	Specificity	Fingerprinting?	Source	Marker type
id1007975	1	11110274	G	A	Saltol	Foreground	Anonymous		Infinium chip	SNP
337593	1	11160425	C	T	Saltol	Foreground	Anonymous		Infinium chip	SNP
id1008171	1	11527750	A	G	Saltol	Foreground	Anonymous		Infinium chip	SNP
353599	1	11576126	G	T	Saltol	Foreground	Anonymous		Infinium chip	SNP
357493	1	11694458	A	G	Saltol	Foreground	Anonymous		Infinium chip	SNP
359469	1	11739003	C	T	Saltol	Foreground	Anonymous		Infinium chip	SNP
6846895	6	27371995	A	G	SCM2	Foreground	Anonymous	Fingerprinting	Infinium chip	SNP
6847831	6	27401748	T	C	SCM2	Foreground	Anonymous		Infinium chip	SNP
6851172	6	27505477	A	C	SCM2	Foreground	Anonymous		Infinium chip	SNP
6855107	6	27609767	C	T	SCM2	Foreground	Anonymous		Infinium chip	SNP
9302663	9	6191930	C	T	Sub1	Foreground	Anonymous	Fingerprinting	Infinium chip	SNP
9305671	9	6257447	C	T	Sub1	Foreground	Anonymous		Infinium chip	SNP
9311693	9	6360984	T	C	Sub1	Foreground	Anonymous		Infinium chip	SNP
9333955	9	6774928	T	C	Sub1	Foreground	Anonymous		Infinium chip	SNP
6766696	6	24875490	G	A	TGW6	Foreground	Anonymous		Infinium chip	SNP
6770053	6	24965643	G	A	TGW6	Foreground	Anonymous		Infinium chip	SNP
6770789	6	24994316	A	G	TGW6	Foreground	Anonymous		Infinium chip	SNP
id6013421	6	25026144	G	T	TGW6	Foreground	Anonymous		Infinium chip	SNP
id6013529	6	25148119	A	G	TGW6	Foreground	Anonymous		Infinium chip	SNP
6776685	6	25185556	A	G	TGW6	Foreground	Anonymous		Infinium chip	SNP
6779108	6	25277863	C	T	TGW6	Foreground	Anonymous		Infinium chip	SNP
6783797	6	25387111	G	A	TGW6	Foreground	Anonymous		Infinium chip	SNP
7768382	7	21998892	G	A	TSV1	Foreground	Anonymous		Infinium chip	SNP
7769958	7	22042832	C	T	TSV1	Foreground	Anonymous		Infinium chip	SNP
7772481	7	22126446	G	A	TSV1	Foreground	Anonymous		Infinium chip	SNP
id7003748	7	22277144	G	A	TSV1	Foreground	Anonymous		Infinium chip	SNP
7780169	7	22381105	A	C	TSV1	Foreground	Anonymous		Infinium chip	SNP
5880441	6	1928403	G	A	Waxy	Foreground	Anonymous		Infinium chip	SNP
5880574	6	1934659	C	T	Waxy	Foreground	Anonymous		Infinium chip	SNP
id8007210	8	26316287	G	A	xa13	Foreground	Anonymous		Infinium chip	SNP

Supplemental Table 4. List of marker positions interrogated for assessing biological accuracy and breeding metrics. All positions are relative to the MSU7 reference genome

Marker name	Chro-mosome	Position	Favourable allele	Unfavourable allele	Target QTL	Marker linkage	Specificity	Fingerprinting?	Source	Marker type
9008641	8	26709228	G	A	xa13	Foreground	Anonymous		Infinium chip	SNP
id8007300	8	26755059	A	C	xa13	Foreground	Anonymous		Infinium chip	SNP
9010484	8	26773740	T	G	xa13	Foreground	Anonymous		Infinium chip	SNP
9011188	8	26805791	C	T	xa13	Foreground	Anonymous		Infinium chip	SNP
9016485	8	26951088	C	A	xa13	Foreground	Anonymous		Infinium chip	SNP
9021271	8	27054037	G	T	xa13	Foreground	Anonymous		Infinium chip	SNP
9030959	8	27300242	G	A	xa13	Foreground	Anonymous		Infinium chip	SNP
id11007903	11	21032445	G	A	Xa21	Foreground	Anonymous		Infinium chip	SNP
11641947	11	21084315	A	G	Xa21	Foreground	Anonymous		Infinium chip	SNP
11649048	11	21213958	A	G	Xa21	Foreground	Anonymous		Infinium chip	SNP
11650133	11	21233762	C	T	Xa21	Foreground	Anonymous		Infinium chip	SNP
11658330	11	21393571	G	T	Xa21	Foreground	Anonymous		Infinium chip	SNP
11660128	11	21415415	C	T	Xa21	Foreground	Anonymous		Infinium chip	SNP
11664079	11	21508416	G	A	Xa21	Foreground	Anonymous		Infinium chip	SNP
id11008193	11	22008834	T	C	Xa23	Foreground	Anonymous		Infinium chip	SNP
id11008214	11	22044151	T	G	Xa23	Foreground	Anonymous		Infinium chip	SNP
11697728	11	22141169	G	A	Xa23	Foreground	Anonymous		Infinium chip	SNP
11697829	11	22144940	G	A	Xa23	Foreground	Anonymous		Infinium chip	SNP
11712665	11	22439562	A	C	Xa23	Foreground	Anonymous		Infinium chip	SNP
11712754	11	22440795	G	A	Xa23	Foreground	Anonymous		Infinium chip	SNP
11713518	11	22453819	T	C	Xa23	Foreground	Anonymous		Infinium chip	SNP
id11008525	11	22546707	A	G	Xa23	Foreground	Anonymous		Infinium chip	SNP
11915122	11	27015384	A	G	Xa4	Background-recomb	Anonymous		Infinium chip	SNP
11920440	11	27163809	C	T	Xa4	Background-recomb	Anonymous		Infinium chip	SNP
11933644	11	27363747	T	C	Xa4	Background-recomb	Anonymous		Infinium chip	SNP
id11010846	11	27387727	A	G	Xa4	Background-recomb	Anonymous		Infinium chip	SNP
11935925	11	27408026	G	A	Xa4	Background-recomb	Anonymous		Infinium chip	SNP
11945545	11	27591935	T	G	Xa4	Foreground	Anonymous		Infinium chip	SNP
11955604	11	27815939	T	G	Xa4	Foreground	Anonymous		Infinium chip	SNP
11955796	11	27817978	C	T	Xa4	Foreground	Anonymous		Infinium chip	SNP

Supplemental Table 4. List of marker positions interrogated for assessing biological accuracy and breeding metrics. All positions are relative to the MSU7 reference genome

Marker name	Chro-mosome	Position	Favourable allele	Unfavourable allele	Target QTL	Marker linkage	Specificity	Fingerprinting?	Source	Marker type
11963686	11	27994133	T	C	Xa4	Foreground	Anonymous		Infinium chip	SNP
11964077	11	27998933	T	C	Xa4	Foreground	Anonymous		Infinium chip	SNP
11977172	11	28312708	A	G	Xa4	Foreground	Anonymous		Infinium chip	SNP
11979043	11	28354517	A	C	Xa4	Foreground	Anonymous		Infinium chip	SNP
11982569	11	28437127	A	G	Xa4	Foreground	Anonymous		Infinium chip	SNP
11985463	11	28485469	A	G	Xa4	Foreground	Anonymous		Infinium chip	SNP
4813168	5	255323	C	T	Xa5	Foreground	Anonymous		Infinium chip	SNP
rd5001427	5	277001	G	A	Xa5	Foreground	Anonymous		Infinium chip	SNP
4814140	5	313688	G	T	Xa5	Foreground	Anonymous		Infinium chip	SNP
4814385	5	327023	A	G	Xa5	Foreground	Anonymous		Infinium chip	SNP
4821710	5	632216	T	C	Xa5	Foreground	Anonymous		Infinium chip	SNP
4822089	5	651569	A	C	Xa5	Foreground	Anonymous		Infinium chip	SNP
4823581	5	686650	G	A	Xa5	Foreground	Anonymous		Infinium chip	SNP
4824019	5	701470	C	T	Xa5	Foreground	Anonymous		Infinium chip	SNP
4827324	5	767585	A	G	Xa5	Foreground	Anonymous		Infinium chip	SNP
6855146	6	27612061	C	T	Xa7	Foreground	Anonymous	Fingerprinting	Infinium chip	SNP
id6015793	6	27627615	A	C	Xa7	Foreground	Anonymous	Fingerprinting	Infinium chip	SNP
6859259	6	27732310	A	G	Xa7	Foreground	Anonymous		Infinium chip	SNP
6859766	6	27761109	C	A	Xa7	Foreground	Anonymous		Infinium chip	SNP
6864051	6	27900453	C	T	Xa7	Foreground	Anonymous		Infinium chip	SNP
6869114	6	28023780	C	A	Xa7	Foreground	Anonymous		Infinium chip	SNP
id6016142	6	28061782	G	A	Xa7	Foreground	Anonymous		Infinium chip	SNP
MSU7_4_6957474_[G/A]	4	6957474	G	A	BPH17	Foreground	Trait-specific	Fingerprinting	Published	SNP
MSU7_4_6979033_[G/C]	4	6979033	G	C	BPH17	Foreground	Trait-specific	Fingerprinting	Published	SNP
Types 5,6,7 (Donors) Pro-1	5	3339934	C	G	Chalk5	Foreground	Trait-specific	Fingerprinting	Published	SNP
COLD1-jap	4	30315214	T	G	COLD1	Foreground	Trait-specific	Fingerprinting	Published	SNP
DEP1-Donor2	9	16415265	-1/-638	0	DEP1	Foreground	Trait-specific	Fingerprinting	Published	Indel
DTH8-IR24	8	4334417	T	-1	DTH8	Foreground	Trait-specific	Fingerprinting	Published	Indel
MSU7_12_17391484_[G/T]	12	17391484	T	G	DTY12.1	Foreground	Trait-specific	Fingerprinting	Published	SNP
BADH2	8	20382857	-8	0	frg-1	Foreground	Trait-specific	Fingerprinting	Published	Indel

Supplemental Table 4. List of marker positions interrogated for assessing biological accuracy and breeding metrics. All positions are relative to the MSU7 reference genome

Marker name	Chro-mosome	Position	Favourable allele	Unfavourable allele	Target QTL	Marker linkage	Specificity	Fingerprinting?	Source	Marker type
MSU7_1_5276521_[T/A]	1	5276521	T	A	Gn1a	Foreground	Trait-specific	Fingerprinting	Published	SNP
MSU7_3_16733441_[T/G]	3	16733441	T	G	GS3	Foreground	Trait-specific	Fingerprinting	Published	SNP
MSU7_6_6752888_[T/C]	6	6752888	T	C	GT - SSIIa	Foreground	Trait-specific	Fingerprinting	Published	SNP
MSU7_2_24771726_[T/A]	2	24771726	T	A	LTG1	Foreground	Trait-specific	Fingerprinting	Published	SNP
PGWC8-2_SNP	8	25894130	G	A	PGWC8-2	Foreground	Trait-specific	Fingerprinting	Published	SNP
PGWC8-2_Indel	8	25897547	0	+10	PGWC8-2	Foreground	Trait-specific		Published	SSR
Pi35_E1054D	1	33144288	C	A	Pi35(t)	Foreground	Trait-specific	Fingerprinting	Published	SNP
MSU7_11_25263936_[Indel]	11	25263936	-34	0	Pi54	Foreground	Trait-specific	Fingerprinting	Published	Indel
MSU7_12_10607554_[C/A]	12	10607554	C	A	Pita2	Foreground	Trait-specific	Fingerprinting	Published	SNP
Xa13-promoter	8	26728873	+~230	0	xa13	Foreground	Trait-specific	Fingerprinting	Published	Indel
MSU7_5_437499_[A/T]	5	437499	A	T	Xa5	Foreground	Trait-specific	Fingerprinting	Published	SNP
MSU7_5_437500_[G/C]	5	437500	G	C	Xa5	Foreground	Trait-specific	Fingerprinting	Published	SNP
MSU7_9_12131787_[Indel]	9	12131787	0	+10	AG1	Foreground	Trait-specific		This work	Indel
MSU7_9_12131957_[G/A]	9	12131957	G	A	AG1	Foreground	Trait-specific	Fingerprinting	This work	SNP
MSU7_9_12132448_[Indel]	9	12132448	-17	0	AG1	Foreground	Trait-specific		This work	Indel
MSU7_9_12154745_[A/C]	9	12154745	A	C	AG1	Foreground	Trait-specific		This work	SNP
MSU7_9_12154800_[Indel]	9	12154800	-20	0	AG1	Foreground	Trait-specific	Fingerprinting	This work	Indel
MSU7_9_12206357_[Indel]	9	12206357	-38	0	AG1	Foreground	Trait-specific	Fingerprinting	This work	Indel
MSU7_9_12207504_[A/G]	9	12207504	A	G	AG1	Foreground	Trait-specific	Fingerprinting	This work	SNP
MSU7_9_12215550_[A/G]	9	12215550	A	G	AG1	Foreground	Trait-specific		This work	SNP
MSU7_9_12281043_[Indel]	9	12281043	0	-30	AG1	Foreground	Trait-specific		This work	Indel
MSU7_9_12333777_[T/C]	9	12333777	C	T	AG1	Foreground	Trait-specific	Fingerprinting	This work	SNP
MSU7_9_12333815_[Indel]	9	12333815	0	-11	AG1	Foreground	Trait-specific	Fingerprinting	This work	Indel
MSU7_4_6933648_[Indel]	4	6933648	-15	0	BPH17	Foreground	Trait-specific		This work	Indel
MSU7_4_6937106_[Indel]	4	6937106	-7	0	BPH17	Foreground	Trait-specific		This work	Indel
MSU7_4_6942449_[T/C]	4	6942449	T	C	BPH17	Foreground	Trait-specific	Fingerprinting	This work	SNP
MSU7_6_1605856_[A/C]	6	1605856	A	C	BPH3	Foreground	Trait-specific	Fingerprinting	This work	SNP
GT 5-2	5	3336278	A	C	Chalk5	Foreground	Trait-specific		This work	SNP
GT 5-1	5	3339314	T	G	Chalk5	Foreground	Trait-specific		This work	SNP
GT 1, 7 Pro-1	5	3339983	A	G	Chalk5	Foreground	Trait-specific		This work	SNP

Supplemental Table 4. List of marker positions interrogated for assessing biological accuracy and breeding metrics. All positions are relative to the MSU7 reference genome

Marker name	Chro-mosome	Position	Favourable allele	Unfavourable allele	Target QTL	Marker linkage	Specificity	Fingerprinting?	Source	Marker type
GT 1,2,3 Pro-1	5	3340202	G	A	Chalk5	Foreground	Trait-specific		This work	SNP
GT 5,6,7 Pro-2	5	3340204	A	T	Chalk5	Foreground	Trait-specific		This work	SNP
GT 5,6,7 Pro-3	5	3340295	G	A	Chalk5	Foreground	Trait-specific		This work	SNP
GT 5,6,7 Pro-4	5	3340440	A	G	Chalk5	Foreground	Trait-specific		This work	SNP
Chalk5_Type-1,7	5	3340698	0	-12	Chalk5	Foreground	Trait-specific		This work	Indel
MSU7_4_29274765_[Indel]	4	29274765	0	-24	COLD1	Background-recomb	Trait-specific		This work	Indel
MSU7_4_30307709_[Indel]	4	30307709	0	-8	COLD1	Foreground	Trait-specific		This work	Indel
MSU7_4_30332386_[Indel]	4	30332386	0	-9	COLD1	Foreground	Trait-specific		This work	Indel
MSU7_4_31162689_[Indel]	4	31162689	0	-17	COLD1	Background-recomb	Trait-specific		This work	Indel
MSU7_9_15278878_[Indel]	9	15278878	0	-8	DEP1	Background-recomb	Trait-specific		This work	Indel
MSU7_9_16364974_[Indel]	9	16364974	0	-11	DEP1	Background-recomb	Trait-specific		This work	Indel
MSU7_9_16417966_[Indel]	9	16417966	0	-11	DEP1	Background-recomb	Trait-specific		This work	Indel
MSU7_9_16948253_[Indel]	9	16948253	0	-14	DEP1	Background-recomb	Trait-specific		This work	Indel
MSU6_Chr01_37924922	1	37925965	G	A	DTY1.1	Foreground	Trait-specific		This work	SNP
MSU7_1_38039503_[Indel]	1	38039503	-24	0	DTY1.1	Foreground	Trait-specific		This work	Indel
MSU7_1_38073598_[Indel]	1	38073598	0	-15	DTY1.1	Foreground	Trait-specific	Fingerprinting	This work	Indel
MSU7_1_38081544_[G/C]	1	38081544	G	C	DTY1.1	Foreground	Trait-specific	Fingerprinting	This work	SNP
MSU6_1_38455686_[C/A]	1	38456729	A	C	DTY1.1	Foreground	Trait-specific		This work	SNP
MSU7_1_38474926_[Indel]	1	38474926	0	-9	DTY1.1	Foreground	Trait-specific	Fingerprinting	This work	Indel
MSU7_1_38652270_[T/C]	1	38652270	T	C	DTY1.1	Foreground	Trait-specific	Fingerprinting	This work	SNP
MSU7_1_38692487_[Indel]	1	38692487	0	-12	DTY1.1	Foreground	Trait-specific	Fingerprinting	This work	Indel
MSU7_1_38843701_[Indel]	1	38843701	-28	0	DTY1.1	Foreground	Trait-specific		This work	Indel
MSU7_1_38997055_[A/C]	1	38997055	A	C	DTY1.1	Foreground	Trait-specific	Fingerprinting	This work	SNP
MSU7_1_39014751_[A/G]	1	39014751	A	G	DTY1.1	Foreground	Trait-specific	Fingerprinting	This work	SNP
MSU7_1_39227750_[G/A]	1	39227750	G	A	DTY1.1	Foreground	Trait-specific		This work	SNP
MSU7_1_39296701_[Indel]	1	39296701	-31	0	DTY1.1	Foreground	Trait-specific		This work	Indel
MSU7_1_39362114_[Indel]	1	39362114	-9	0	DTY1.1	Foreground	Trait-specific		This work	Indel
MSU7_1_39534368_[G/A]	1	39534368	G	A	DTY1.1	Foreground	Trait-specific	Fingerprinting	This work	SNP
MSU7_1_39544672_[G/C]	1	39544672	C	G	DTY1.1	Foreground	Trait-specific		This work	SNP
MSU7_1_39610271_[G/T]	1	39610271	G	T	DTY1.1	Foreground	Trait-specific	Fingerprinting	This work	SNP

Supplemental Table 4. List of marker positions interrogated for assessing biological accuracy and breeding metrics. All positions are relative to the MSU7 reference genome

Marker name	Chro-mosome	Position	Favourable allele	Unfavourable allele	Target QTL	Marker linkage	Specificity	Fingerprinting?	Source	Marker type
MSU7_1_39799661_[Indel]	1	39799661	-15	0	DTY1.1	Foreground	Trait-specific		This work	Indel
MSU7_1_40467064_[T/C]	1	40467064	C	T	DTY1.1	Foreground	Trait-specific		This work	SNP
MSU7_12_17354715_[Indel]	12	17354715	-8	0	DTY12.1	Foreground	Trait-specific	Fingerprinting	This work	Indel
MSU7_12_17391872_[A/G]	12	17391872	A	G	DTY12.1	Foreground	Trait-specific	Fingerprinting	This work	SNP
MSU7_12_17410887_[Indel]	12	17410887	-18	0	DTY12.1	Foreground	Trait-specific	Fingerprinting	This work	Indel
MSU7_2_9600368_[C/T]	2	9600368	C	T	DTY2.1	Foreground	Trait-specific	Fingerprinting	This work	SNP
MSU7_2_9851737_[A/C]	2	9851737	A	C	DTY2.1	Foreground	Trait-specific	Fingerprinting	This work	SNP
MSU7_2_9892764_[G/A]	2	9892764	G	A	DTY2.1	Foreground	Trait-specific	Fingerprinting	This work	SNP
MSU7_2_10003379_[C/A]	2	10003379	C	A	DTY2.1	Foreground	Trait-specific	Fingerprinting	This work	SNP
MSU7_2_10010893_[T/C]	2	10010893	C	T	DTY2.1	Foreground	Trait-specific	Fingerprinting	This work	SNP
MSU7_2_10077216_[G/T]	2	10077216	T	G	DTY2.1	Foreground	Trait-specific	Fingerprinting	This work	SNP
MSU7_2_10182155_[T/C]	2	10182155	C	T	DTY2.1	Foreground	Trait-specific	Fingerprinting	This work	SNP
MSU7_2_10183246_[A/G]	2	10183246	A	G	DTY2.1	Foreground	Trait-specific	Fingerprinting	This work	SNP
MSU7_2_10377052_[A/G]	2	10377052	A	G	DTY2.1	Foreground	Trait-specific	Fingerprinting	This work	SNP
MSU7_2_10640630_[A/G]	2	10640630	G	A	DTY2.1	Foreground	Trait-specific	Fingerprinting	This work	SNP
MSU7_2_10747561_[A/G]	2	10747561	A	G	DTY2.1	Foreground	Trait-specific	Fingerprinting	This work	SNP
MSU7_2_10753821_[A/G]	2	10753821	G	A	DTY2.1	Foreground	Trait-specific	Fingerprinting	This work	SNP
MSU7_2_11015895_[T/A]	2	11015895	A	T	DTY2.1	Foreground	Trait-specific	Fingerprinting	This work	SNP
MSU7_2_11152228_[Indel]	2	11152228	-10	0	DTY2.1	Foreground	Trait-specific	Fingerprinting	This work	Indel
MSU7_2_11153055_[A/T]	2	11153055	A	T	DTY2.1	Foreground	Trait-specific	Fingerprinting	This work	SNP
MSU7_2_11211992_[T/C]	2	11211992	T	C	DTY2.1	Foreground	Trait-specific	Fingerprinting	This work	SNP
MSU7_2_12020819_[C/T]	2	12020819	C	T	DTY2.1	Foreground	Trait-specific		This work	SNP
MSU6_2_209765_[G/A]	2	209765	G	A	DTY2.2	Foreground	Trait-specific		This work	SNP
MSU6_2_973068_[Indel]	2	973068	-14	0	DTY2.2	Foreground	Trait-specific		This work	Indel
MSU7_2_1660713_[C/T]	2	1660713	T	C	DTY2.2	Foreground	Trait-specific		This work	SNP
MSU7_2_1678460_[G/A]	2	1678460	G	A	DTY2.2	Foreground	Trait-specific		This work	SNP
MSU6_2_2073485_[A/C]	2	2073485	C	A	DTY2.2	Foreground	Trait-specific		This work	SNP
MSU7_2_2085377_[G/C]	2	2085377	G	C	DTY2.2	Foreground	Trait-specific		This work	SNP
MSU7_2_2092536_[C/T]	2	2092536	C	T	DTY2.2	Foreground	Trait-specific		This work	SNP
MSU6_2_2394200_[Indel]	2	2394202	0	+11	DTY2.2	Foreground	Trait-specific		This work	Indel

Supplemental Table 4. List of marker positions interrogated for assessing biological accuracy and breeding metrics. All positions are relative to the MSU7 reference genome

Marker name	Chro-mosome	Position	Favourable allele	Unfavourable allele	Target QTL	Marker linkage	Specificity	Fingerprinting?	Source	Marker type
MSU7_2_2542883_[T/A]	2	2542883	T	A	DTY2.2	Foreground	Trait-specific	Fingerprinting	This work	SNP
MSU6_2_2560207_[C/T]	2	2560209	T	C	DTY2.2	Foreground	Trait-specific		This work	SNP
MSU6_2_2647007_[Indel]	2	2647009	-8	0	DTY2.2	Foreground	Trait-specific		This work	Indel
MSU6_2_2777797_[C/T]	2	2777799	T	C	DTY2.2	Foreground	Trait-specific		This work	SNP
MSU6_2_2792378_[G/A]	2	2792380	A	G	DTY2.2	Foreground	Trait-specific		This work	SNP
MSU7_2_3029440_[T/C]	2	3029440	T	C	DTY2.2	Foreground	Trait-specific	Fingerprinting	This work	SNP
MSU6_2_3192246_[Indel]	2	3192248	-53	0	DTY2.2	Foreground	Trait-specific		This work	Indel
MSU6_2_3200037_[G/A]	2	3200039	A	G	DTY2.2	Foreground	Trait-specific		This work	SNP
MSU6_2_3202243_[Indel]	2	3202245	-9	0	DTY2.2	Foreground	Trait-specific		This work	Indel
MSU7_2_3597626_[G/C]	2	3597626	G	C	DTY2.2	Foreground	Trait-specific	Fingerprinting	This work	SNP
MSU6_2_3835922_[A/C]	2	3835925	C	A	DTY2.2	Foreground	Trait-specific		This work	SNP
MSU6_2_3949702_[Indel]	2	3949705	0	-13	DTY2.2	Foreground	Trait-specific		This work	Indel
MSU7_2_4020520_[Indel]	2	4020520	0	-23	DTY2.2	Foreground	Trait-specific	Fingerprinting	This work	Indel
MSU7_2_4038100_[A/T]	2	4038100	A	T	DTY2.2	Foreground	Trait-specific		This work	SNP
MSU7_2_4044509_[A/C]	2	4044509	A	C	DTY2.2	Foreground	Trait-specific		This work	SNP
MSU6_2_4316330_[T/G]	2	4316333	T	G	DTY2.2	Foreground	Trait-specific		This work	SNP
MSU7_2_4565707_[T/C]	2	4565707	T	C	DTY2.2	Foreground	Trait-specific		This work	SNP
MSU6_2_4871843_[A/G]	2	4871846	A	G	DTY2.2	Foreground	Trait-specific	Fingerprinting	This work	SNP
MSU6_2_4903393_[C/A]	2	4903396	A	C	DTY2.2	Foreground	Trait-specific		This work	SNP
MSU7_2_4943142_[Indel]	2	4943142	0	-21	DTY2.2	Foreground	Trait-specific		This work	Indel
MSU7_2_5019293_[T/A]	2	5019293	T	A	DTY2.2	Foreground	Trait-specific		This work	SNP
MSU7_2_5021486_[T/C]	2	5021486	T	C	DTY2.2	Foreground	Trait-specific		This work	SNP
MSU6_2_5238637_[G/A]	2	5238640	A	G	DTY2.2	Foreground	Trait-specific	Fingerprinting	This work	SNP
MSU6_2_5241905_[Indel]	2	5241908	-13	0	DTY2.2	Foreground	Trait-specific	Fingerprinting	This work	Indel
MSU6_2_5275139_[G/A]	2	5275142	A	G	DTY2.2	Foreground	Trait-specific		This work	SNP
MSU6_2_5279054_[T/C]	2	5279057	C	T	DTY2.2	Foreground	Trait-specific	Fingerprinting	This work	SNP
MSU6_2_5280764_[Indel]	2	5280767	0	-24	DTY2.2	Foreground	Trait-specific		This work	Indel
MSU7_2_5501035_[Indel]	2	5501035	-10	0	DTY2.2	Foreground	Trait-specific		This work	SNP
MSU7_2_5504975_[G/A]	2	5504975	G	A	DTY2.2	Foreground	Trait-specific		This work	SNP
MSU7_2_5515259_[G/T]	2	5515259	G	T	DTY2.2	Foreground	Trait-specific	Fingerprinting	This work	SNP

Supplemental Table 4. List of marker positions interrogated for assessing biological accuracy and breeding metrics. All positions are relative to the MSU7 reference genome

Marker name	Chro-mosome	Position	Favourable allele	Unfavourable allele	Target QTL	Marker linkage	Specificity	Fingerprinting?	Source	Marker type
MSU6_2_5688475_[G/A]	2	5688475	A	G	DTY2.2	Foreground	Trait-specific	Fingerprinting	This work	SNP
MSU6_2_5689478_[G/A]	2	5689478	A	G	DTY2.2	Foreground	Trait-specific		This work	SNP
MSU6_2_5694476_[Indel]	2	5694476	0	-10	DTY2.2	Foreground	Trait-specific		This work	Indel
MSU7_2_6009221_[G/T]	2	6009221	G	T	DTY2.2	Foreground	Trait-specific		This work	SNP
MSU7_2_6267235_[C/T]	2	6267235	C	T	DTY2.2	Foreground	Trait-specific	Fingerprinting	This work	SNP
MSU6_2_6587603_[T/G]	2	6587603	T	G	DTY2.2	Foreground	Trait-specific		This work	SNP
MSU7_2_6623323_[G/A]	2	6623323	G	A	DTY2.2	Foreground	Trait-specific	Fingerprinting	This work	SNP
MSU7_2_7005817_[T/G]	2	7005817	T	G	DTY2.2	Foreground	Trait-specific		This work	SNP
MSU6_2_7485339_[C/T]	2	7485339	T	C	DTY2.2	Foreground	Trait-specific	Fingerprinting	This work	SNP
MSU6_2_7499561_[Indel]	2	7499561	-10	0	DTY2.2	Foreground	Trait-specific	Fingerprinting	This work	Indel
MSU6_2_8594377_[Indel]	2	8594378	0	-21	DTY2.2	Foreground	Trait-specific		This work	Indel
MSU6_2_8882314_[Indel]	2	8882316	-9	0	DTY2.2	Foreground	Trait-specific		This work	Indel
MSU6_3_28587948_[G/A]	3	28587948	G	A	DTY3.1	Foreground	Trait-specific	Fingerprinting	This work	SNP
MSU7_3_30047928_[G/A]	3	30047928	G	A	DTY3.1	Foreground	Trait-specific	Fingerprinting	This work	SNP
MSU7_3_30736463_[G/A]	3	30736463	G	A	DTY3.1	Foreground	Trait-specific	Fingerprinting	This work	SNP
MSU7_3_30738342_[C/G]	3	30738342	C	G	DTY3.1	Foreground	Trait-specific	Fingerprinting	This work	SNP
MSU7_3_30742072_[T/G]	3	30742072	T	G	DTY3.1	Foreground	Trait-specific	Fingerprinting	This work	SNP
MSU7_3_30747956_[T/C]	3	30747956	T	C	DTY3.1	Foreground	Trait-specific	Fingerprinting	This work	SNP
MSU7_3_30807050_[G/A]	3	30807050	G	A	DTY3.1	Foreground	Trait-specific	Fingerprinting	This work	SNP
3_30851172	3	30858283	-17	0	DTY3.1	Foreground	Trait-specific	Fingerprinting	This work	Indel
MSU7_3_30931067_[C/G]	3	30931067	C	G	DTY3.1	Foreground	Trait-specific	Fingerprinting	This work	SNP
MSU7_3_30958327_[G/A]	3	30958327	G	A	DTY3.1	Foreground	Trait-specific	Fingerprinting	This work	SNP
MSU7_3_30960435_[A/G]	3	30960435	A	G	DTY3.1	Foreground	Trait-specific	Fingerprinting	This work	SNP
MSU7_3_30961142_[A/G]	3	30961142	A	G	DTY3.1	Foreground	Trait-specific	Fingerprinting	This work	SNP
MSU7_3_31008659_[C/T]	3	31008659	C	T	DTY3.1	Foreground	Trait-specific	Fingerprinting	This work	SNP
MSU7_3_31016149_[A/G]	3	31016149	A	G	DTY3.1	Foreground	Trait-specific	Fingerprinting	This work	SNP
MSU7_3_31017207_[T/C]	3	31017207	T	C	DTY3.1	Foreground	Trait-specific	Fingerprinting	This work	SNP
MSU7_3_31041777_[A/T]	3	31041777	A	T	DTY3.1	Foreground	Trait-specific	Fingerprinting	This work	SNP
MSU7_3_31050704_[A/G]	3	31050704	A	G	DTY3.1	Foreground	Trait-specific	Fingerprinting	This work	SNP
MSU7_3_31323659_[C/G]	3	31323659	C	G	DTY3.1	Foreground	Trait-specific	Fingerprinting	This work	SNP

Supplemental Table 4. List of marker positions interrogated for assessing biological accuracy and breeding metrics. All positions are relative to the MSU7 reference genome

Marker name	Chro-mosome	Position	Favourable allele	Unfavourable allele	Target QTL	Marker linkage	Specificity	Fingerprinting?	Source	Marker type
MSU7_3_31344952_[T/C]	3	31344952	T	C	DTY3.1	Foreground	Trait-specific	Fingerprinting	This work	SNP
MSU6_3_190234_[A/G]	3	191234	A	G	DTY3.2	Foreground	Trait-specific		This work	SNP
MSU6_3_798667_[InDel]	3	799669	-23	0	DTY3.2	Foreground	Trait-specific		This work	Indel
MSU6_3_804250_[InDel]	3	805252	+7	0	DTY3.2	Foreground	Trait-specific		This work	Indel
MSU6_3_807375_[T/C]	3	808377	C	T	DTY3.2	Foreground	Trait-specific		This work	SNP
MSU6_3_1292055_[InDel]	3	1293057	0	-10	DTY3.2	Foreground	Trait-specific	Fingerprinting	This work	Indel
MSU6_3_1293822_[InDel]	3	1294824	-53	-9	DTY3.2	Foreground	Trait-specific	Fingerprinting	This work	Indel
MSU7_3_1295945_[A/G]	3	1295945	G	A	DTY3.2	Foreground	Trait-specific	Fingerprinting	This work	SNP
MSU6_3_1296879_[InDel]	3	1296879	-29	0	DTY3.2	Foreground	Trait-specific		This work	Indel
MSU7_3_1297998_[G/A]	3	1297998	G	A	DTY3.2	Foreground	Trait-specific		This work	SNP
MSU6_3_1829636_[C/T]	3	1830640	C	T	DTY3.2	Foreground	Trait-specific		This work	SNP
MSU6_4_89279_[A/T]	4	90279	A	T	DTY4.1	Foreground	Trait-specific	Fingerprinting	This work	SNP
MSU6_4_91491_[G/T]	4	92491	G	T	DTY4.1	Foreground	Trait-specific	Fingerprinting	This work	SNP
MSU6_4_174984_[C/T]	4	175984	T	C	DTY4.1	Foreground	Trait-specific	Fingerprinting	This work	SNP
MSU6_4_196102_[Indel]	4	197102	-9	0	DTY4.1	Foreground	Trait-specific	Fingerprinting	This work	Indel
MSU6_4_221034_[T/C]	4	222034	C	T	DTY4.1	Foreground	Trait-specific	Fingerprinting	This work	SNP
MSU6_4_329380_[T/C]	4	330380	C	T	DTY4.1	Foreground	Trait-specific	Fingerprinting	This work	SNP
MSU6_4_344064_[T/C]	4	345064	C	T	DTY4.1	Foreground	Trait-specific	Fingerprinting	This work	SNP
MSU6_4_482253_[Indel]	4	483255	-32	0	DTY4.1	Foreground	Trait-specific	Fingerprinting	This work	Indel
MSU6_4_483827_[G/T]	4	484829	G	T	DTY4.1	Foreground	Trait-specific	Fingerprinting	This work	SNP
MSU6_4_913761_[A/C]	4	914761	C	A	DTY4.1	Foreground	Trait-specific	Fingerprinting	This work	SNP
MSU6_4_919402_[C/A]	4	920402	A	C	DTY4.1	Foreground	Trait-specific		This work	SNP
MSU6_4_1068894_[Indel]	4	1073343	-10	0	DTY4.1	Foreground	Trait-specific	Fingerprinting	This work	Indel
MSU6_4_1146119_[C/T]	4	1150569	T	C	DTY4.1	Foreground	Trait-specific	Fingerprinting	This work	SNP
MSU6_4_1329641_[Indel]	4	1334081	-20	0	DTY4.1	Foreground	Trait-specific	Fingerprinting	This work	Indel
MSU6_4_1750084_[Indel]	4	1754486	0	-12	DTY4.1	Foreground	Trait-specific	Fingerprinting	This work	Indel
MSU6_4_1965257_[T/C]	4	1969661	C	T	DTY4.1	Foreground	Trait-specific	Fingerprinting	This work	SNP
MSU6_4_1977984_[indel]	4	1982388	-10	0	DTY4.1	Foreground	Trait-specific	Fingerprinting	This work	Indel
MSU6_4_2277564_[C/T]	4	2281969	T	C	DTY4.1	Foreground	Trait-specific	Fingerprinting	This work	SNP
MSU6_4_2281606_[Indel]	4	2286011	-13	0	DTY4.1	Foreground	Trait-specific	Fingerprinting	This work	Indel

Supplemental Table 4. List of marker positions interrogated for assessing biological accuracy and breeding metrics. All positions are relative to the MSU7 reference genome

Marker name	Chro-mosome	Position	Favourable allele	Unfavourable allele	Target QTL	Marker linkage	Specificity	Fingerprinting?	Source	Marker type
MSU6_4_2404881_[C/T]	4	2409286	T	C	DTY4.1	Foreground	Trait-specific	Fingerprinting	This work	SNP
MSU6_4_3553389_[G/A]	4	3557787	A	G	DTY4.1	Foreground	Trait-specific		This work	SNP
MSU7_8_20383435_[T/C]	8	20383435	T	C	frg-1	Foreground	Trait-specific		This work	SNP
MSU7_1_4949689_[Indel]	1	4949689	0	-20	Gn1a	Foreground	Trait-specific		This work	Indel
MSU7_1_5210929_[Indel]	1	5210929	-30	0	Gn1a	Foreground	Trait-specific		This work	Indel
MSU7_1_5413105_[Indel]	1	5413105	-18	0	Gn1a	Foreground	Trait-specific		This work	Indel
MSU7_1_5545332_[Indel]	1	5545332	-28	0	Gn1a	Foreground	Trait-specific		This work	Indel
MSU7_1_5653351_[Indel]	1	5653351	-11	0	Gn1a	Background-recomb	Trait-specific		This work	Indel
MSU7_3_16731553_[Indel]	3	16731553	0	-14	GS3	Foreground	Trait-specific		This work	Indel
MSU7_3_16733632_[Indel]	3	16733632	0	+45	GS3	Foreground	Trait-specific		This work	Indel
MSU7_6_6747818_[Indel]	6	6747818	0	-9	GT - SSIIa	Foreground	Trait-specific		This work	Indel
MSU7_6_6748824_[C/G]	6	6748824	C	G	GT - SSIIa	Foreground	Trait-specific		This work	SNP
MSU7_6_6752357_[A/G]	6	6752357	A	G	GT - SSIIa	Foreground	Trait-specific		This work	SNP
MSU7_6_6752756_[A/G]	6	6752756	A	G	GT - SSIIa	Foreground	Trait-specific		This work	SNP
MSU7_6_6754433_[Indel]	6	6754433	0	-14	GT - SSIIa	Foreground	Trait-specific		This work	Indel
MSU7_6_6763950_[Indel]	6	6763950	0	-18	GT - SSIIa	Foreground	Trait-specific		This work	Indel
MSU7_5_5354753_[Indel]	5	5354753	-29	0	GW5/SW5	Foreground	Trait-specific	Fingerprinting	This work	Indel
MSU7_5_5359425_[A/G]	5	5359425	A	G	GW5/SW5	Foreground	Trait-specific		This work	SNP
MSU7_5_5360896_[T/C]	5	5360896	T	C	GW5/SW5	Foreground	Trait-specific		This work	SNP
MSU7_5_5361329_[A/G]	5	5361329	A	G	GW5/SW5	Foreground	Trait-specific		This work	SNP
MSU7_5_5361420_[Indel]	5	5361420	-36	0	GW5/SW5	Foreground	Trait-specific		This work	Indel
3_1011486	3	1012487	-10	0	Hd9	Foreground	Trait-specific		This work	Indel
MSU7_3_1270943_[A/G]	3	1270943	G	A	Hd9	Foreground	Trait-specific	Fingerprinting	This work	SNP
MSU7_3_1271083_[C/T]	3	1271083	C	T	Hd9	Foreground	Trait-specific		This work	SNP
MSU6_3_1270081_[T/C]	3	1271083	C	T	Hd9	Foreground	Trait-specific		This work	SNP
MSU7_3_1271431_[A/G]	3	1271431	A	G	Hd9	Foreground	Trait-specific		This work	SNP
MSU6_3_1270666_[G/C]	3	1271668	G	C	Hd9	Foreground	Trait-specific		This work	SNP
MSU7_3_1290074_[T/G]	3	1290074	T	G	Hd9	Foreground	Trait-specific		This work	SNP
MSU6_3_1289949_[InDel]	3	1290947	-16	0	Hd9	Foreground	Trait-specific		This work	Indel
3_1850409	3	1851409	-16	0	Hd9	Foreground	Trait-specific		This work	Indel

Supplemental Table 4. List of marker positions interrogated for assessing biological accuracy and breeding metrics. All positions are relative to the MSU7 reference genome

Marker name	Chro-mosome	Position	Favourable allele	Unfavourable allele	Target QTL	Marker linkage	Specificity	Fingerprinting?	Source	Marker type
MSU6_4_17302271_[Indel]	4	17474229	0	-22	HTSF4.1	Foreground	Trait-specific	Fingerprinting	This work	Indel
MSU6_4_17306068_[Indel]	4	17478038	0	+17	HTSF4.1	Foreground	Trait-specific	Fingerprinting	This work	SSR
MSU6_4_17313683_[Indel]	4	17485641	0	-11	HTSF4.1	Foreground	Trait-specific		This work	Indel
MSU6_4_17414046_[Indel]	4	17586003	-17	0	HTSF4.1	Foreground	Trait-specific		This work	Indel
MSU6_4_17432397_[Indel]	4	17604356	0	+34	HTSF4.1	Foreground	Trait-specific	Fingerprinting	This work	Indel
MSU6_4_17434648_[Indel]	4	17606607	+>30	0	HTSF4.1	Foreground	Trait-specific		This work	Indel
MSU6_4_17482836_[Indel]	4	17654795	-83	0	HTSF4.1	Foreground	Trait-specific	Fingerprinting	This work	Indel
MSU6_4_17485204_[Indel]	4	17657163	-27	0	HTSF4.1	Foreground	Trait-specific		This work	Indel
MSU6_4_17495280_[Indel]	4	17667229	0	+15	HTSF4.1	Foreground	Trait-specific	Fingerprinting	This work	Indel
Heat_85	4	17671097	A	T	HTSF4.1	Foreground	Trait-specific	Fingerprinting	This work	SNP
MSU6_4_17719426_[Indel]	4	17891385	0	-15	HTSF4.1	Foreground	Trait-specific	Fingerprinting	This work	Indel
MSU6_4_17987548_[Indel]	4	18159482	0	-22	HTSF4.1	Foreground	Trait-specific	Fingerprinting	This work	Indel
MSU6_4_17996441_[Indel]	4	18168397	0	+>40	HTSF4.1	Foreground	Trait-specific	Fingerprinting	This work	Indel
Heat_88	4	18172991	C	G	HTSF4.1	Foreground	Trait-specific	Fingerprinting	This work	SNP
MSU6_4_18081825_[Indel]	4	18253779	0	+11	HTSF4.1	Foreground	Trait-specific		This work	Indel
MSU6_4_18231862_[Indel]	4	18403817	0	-42	HTSF4.1	Foreground	Trait-specific		This work	Indel
MSU6_4_18241283_[Indel]	4	18413238	0	-7	HTSF4.1	Foreground	Trait-specific	Fingerprinting	This work	Indel
MSU6_4_18349442_[Indel]	4	18521397	-16	0	HTSF4.1	Foreground	Trait-specific		This work	Indel
MSU6_4_18353408_[Indel]	4	18525363	0	-8	HTSF4.1	Foreground	Trait-specific	Fingerprinting	This work	Indel
MSU6_4_18434206_[Indel]	4	18606160	-34	0	HTSF4.1	Foreground	Trait-specific	Fingerprinting	This work	Indel
Heat_81	4	18775374	G	A	HTSF4.1	Foreground	Trait-specific		This work	SNP
MSU6_4_19012894_[Indel]	4	19184854	0	-17	HTSF4.1	Foreground	Trait-specific		This work	Indel
MSU6_4_19049581_[Indel]	4	19221545	0	-11	HTSF4.1	Foreground	Trait-specific		This work	Indel
MSU6_4_19431105_[Indel]	4	19603067	0	-20	HTSF4.1	Foreground	Trait-specific		This work	Indel
MSU6_4_19698160_[Indel]	4	19870124	-8	0	HTSF4.1	Foreground	Trait-specific		This work	Indel
MSU6_4_19700450_[Indel]	4	19872414	0	+21	HTSF4.1	Foreground	Trait-specific		This work	Indel
MSU6_4_19713054_[Indel]	4	19885018	-4	0	HTSF4.1	Foreground	Trait-specific		This work	SSR
MSU6_4_19713669_[Indel]	4	19885633	0	-220	HTSF4.1	Foreground	Trait-specific		This work	Indel
MSU6_4_19744053_[Indel]	4	19916017	0	+26	HTSF4.1	Foreground	Trait-specific		This work	Indel
MSU6_4_19968600_[Indel]	4	20140560	0	-7	HTSF4.1	Foreground	Trait-specific		This work	Indel

Supplemental Table 4. List of marker positions interrogated for assessing biological accuracy and breeding metrics. All positions are relative to the MSU7 reference genome

Marker name	Chro-mosome	Position	Favourable allele	Unfavourable allele	Target QTL	Marker linkage	Specificity	Fingerprinting?	Source	Marker type
MSU6_4_19977796_[Indel]	4	20149751	0	-24	HTSF4.1	Foreground	Trait-specific		This work	Indel
MSU7_2_24735311_[Indel]	2	24735311	0	-18	LTG1	Foreground	Trait-specific	Fingerprinting	This work	Indel
MSU7_4_30376012_[Indel]	4	30376012	0	-22	NAL1	Foreground	Trait-specific		This work	Indel
MSU6_4_31027691[G/A]	4	31212801	A	G	NAL1	Foreground	Trait-specific	Fingerprinting	This work	SNP
MSU7_4_31358376_[Indel]	4	31358376	0	-11	NAL1	Background-recomb	Trait-specific		This work	Indel
MSU7_8_24789321_[Indel]	8	24789321	0	-14	PGWC8-2	Background-recomb	Trait-specific		This work	Indel
MSU7_8_25891190_[Indel]	8	25891190	0	-14	PGWC8-2	Foreground	Trait-specific		This work	Indel
MSU7_8_25901566_[Indel]	8	25901566	0	-10	PGWC8-2	Foreground	Trait-specific		This work	Indel
MSU7_8_25977796_[Indel]	8	25977796	0	-11	PGWC8-2	Foreground	Trait-specific		This work	Indel
MSU7_8_26171338_[Indel]	8	26171338	0	-27	PGWC8-2	Background-recomb	Trait-specific		This work	Indel
MSU7_1_32975144_[Indel]	1	32975144	0	-21	Pi35(t)	Foreground	Trait-specific		This work	Indel
MSU7_1_33075210_[Indel]	1	33075210	0	-6	Pi35(t)	Foreground	Trait-specific		This work	Indel
Pi35_R1056S	1	33144294	T	A	Pi35(t)	Foreground	Trait-specific		This work	SNP
Pi35_Q1073P	1	33144344	C	A	Pi35(t)	Foreground	Trait-specific		This work	SNP
MSU7_1_33145816_[Indel]	1	33145816	0	-7	Pi35(t)	Foreground	Trait-specific		This work	Indel
MSU7_1_33177564_[Indel]	1	33177564	+>50	0	Pi35(t)	Foreground	Trait-specific		This work	Indel
MSU7_1_33182396_[Indel]	1	33182396	0	-15	Pi35(t)	Foreground	Trait-specific		This work	Indel
MSU7_1_33202058_[Indel]	1	33202058	-9	0	Pi35(t)	Foreground	Trait-specific		This work	Indel
MSU7_1_33246278_[Indel]	1	33246278	+13	0	Pi35(t)	Foreground	Trait-specific		This work	Indel
MSU7_1_33260985_[Indel]	1	33260985	0	-11	Pi35(t)	Foreground	Trait-specific		This work	Indel
MSU7_11_24998256_[C/T]	11	24998256	C	T	Pi54	Background-recomb	Trait-specific		This work	SNP
MSU7_11_25021561_[C/T]	11	25021561	C	T	Pi54	Background-recomb	Trait-specific		This work	SNP
MSU7_11_25040709_[Indel]	11	25040709	0	-10	Pi54	Background-recomb	Trait-specific		This work	Indel
MSU7_11_25040957_[G/T]	11	25040957	G	T	Pi54	Background-recomb	Trait-specific		This work	SNP
MSU7_11_25121364_[Indel]	11	25121364	0	-38	Pi54	Background-recomb	Trait-specific		This work	Indel
MSU7_11_25160969_[T/C]	11	25160969	T	C	Pi54	Foreground	Trait-specific		This work	SNP
MSU7_11_25161698_[G/A]	11	25161698	G	A	Pi54	Foreground	Trait-specific		This work	SNP
MSU7_11_25174345_[Indel]	11	25174345	-10	0	Pi54	Foreground	Trait-specific		This work	Indel
MSU7_11_25194916_[Indel]	11	25194916	0	-9	Pi54	Foreground	Trait-specific		This work	Indel
MSU7_11_25203227_[Indel]	11	25203227	0	-10	Pi54	Foreground	Trait-specific		This work	Indel

Supplemental Table 4. List of marker positions interrogated for assessing biological accuracy and breeding metrics. All positions are relative to the MSU7 reference genome

Marker name	Chro-mosome	Position	Favourable allele	Unfavourable allele	Target QTL	Marker linkage	Specificity	Fingerprinting?	Source	Marker type
MSU7_11_25203532_[A/T]	11	25203532	A	T	Pi54	Foreground	Trait-specific		This work	SNP
MSU7_11_25208829_[Indel]	11	25208829	-10	0	Pi54	Foreground	Trait-specific		This work	Indel
MSU7_11_25264060_[G/A]	11	25264060	G	A	Pi54	Foreground	Trait-specific		This work	SNP
MSU7_11_25400634_[Indel]	11	25400634	0	-21	Pi54	Foreground	Trait-specific		This work	Indel
MSU7_11_25478473_[Indel]	11	25478473	-12	0	Pi54	Foreground	Trait-specific		This work	Indel
MSU7_11_25495115_[Indel]	11	25495115	0	-13	Pi54	Foreground	Trait-specific		This work	Indel
MSU7_11_25517720_[Indel]	11	25517720	-7	0	Pi54	Foreground	Trait-specific		This work	Indel
MSU7_11_25550877_[Indel]	11	25550877	+30	0	Pi54	Background-recomb	Trait-specific		This work	Indel
MSU7_11_25566130_[Indel]	11	25566130	-15	0	Pi54	Background-recomb	Trait-specific		This work	Indel
MSU7_11_25592940_[Indel]	11	25592940	-27	0	Pi54	Background-recomb	Trait-specific		This work	Indel
MSU7_11_25594012_[Indel]	11	25594012	+18	0	Pi54	Background-recomb	Trait-specific		This work	Indel
MSU7_11_25600423_[Indel]	11	25600423	-8	0	Pi54	Background-recomb	Trait-specific		This work	Indel
MSU7_11_26311147_[G/T]	11	26311147	G	T	Pi54	Background-recomb	Trait-specific		This work	SNP
MSU7_6_10198122_[A/C]	6	10198122	C	A	Pi9	Background-recomb	Trait-specific		This work	SNP
MSU7_6_10381396_[A/G]	6	10381396	A	G	Pi9	Foreground	Trait-specific		This work	SNP
MSU7_6_10381489_[Indel]	6	10381489	-10	0	Pi9	Foreground	Trait-specific	Fingerprinting	This work	Indel
MSU7_6_10381570_[A/C]	6	10381570	A	C	Pi9	Foreground	Trait-specific		This work	SNP
MSU7_6_10381589_[T/C]	6	10381589	T	C	Pi9	Foreground	Trait-specific		This work	SNP
MSU7_6_10381880_[C/T]	6	10381880	C	T	Pi9	Foreground	Trait-specific		This work	SNP
MSU7_6_10383378_[C/T]	6	10383378	T	C	Pi9	Foreground	Trait-specific		This work	SNP
MSU7_6_10388025_[T/A]	6	10388025	T	A	Pi9	Foreground	Trait-specific		This work	SNP
MSU7_6_10389274_[A/C]	6	10389274	A	C	Pi9	Foreground	Trait-specific		This work	SNP
MSU7_6_10389352_[A/T]	6	10389352	A	T	Pi9	Foreground	Trait-specific		This work	SNP
MSU7_6_10389610_[C/G]	6	10389610	C	G	Pi9	Foreground	Trait-specific		This work	SNP
MSU7_6_10389633_[C/G]	6	10389633	C	G	Pi9	Foreground	Trait-specific		This work	SNP
MSU7_6_10390099_[C/A]	6	10390099	C	A	Pi9	Foreground	Trait-specific		This work	SNP
MSU7_6_10711621_[A/T]	6	10711621	A	T	Pi9	Background-recomb	Trait-specific		This work	SNP
MSU7_6_10807099_[T/C]	6	10807099	T	C	Pi9	Background-recomb	Trait-specific		This work	SNP
MSU7_6_10960628_[C/T]	6	10960628	C	T	Pi9	Background-recomb	Trait-specific		This work	SNP
MSU7_12_9177624_[Indel]	12	9177624	-26	0	Pita2	Foreground	Trait-specific		This work	SNP

Supplemental Table 4. List of marker positions interrogated for assessing biological accuracy and breeding metrics. All positions are relative to the MSU7 reference genome

Marker name	Chro-mosome	Position	Favourable allele	Unfavourable allele	Target QTL	Marker linkage	Specificity	Fingerprinting?	Source	Marker type
MSU7_12_9893146_[A/G]	12	9893146	A	G	Pita2	Foreground	Trait-specific		This work	SNP
MSU7_12_9996103_[Indel]	12	9996103	+>40	0	Pita2	Foreground	Trait-specific		This work	Indel
MSU7_12_10177640_[A/G]	12	10177640	A	G	Pita2	Foreground	Trait-specific		This work	SNP
MSU7_12_10188917_[Indel]	12	10188917	-9	0	Pita2	Foreground	Trait-specific		This work	Indel
MSU7_12_10302339_[Indel]	12	10302339	+21	0	Pita2	Foreground	Trait-specific		This work	Indel
MSU7_12_10423020_[T/C]	12	10423020	C	T	Pita2	Foreground	Trait-specific		This work	SNP
MSU7_12_10451426_[Indel]	12	10451426	-21	0	Pita2	Foreground	Trait-specific		This work	Indel
MSU7_12_10508210_[Indel]	12	10508210	-23	0	Pita2	Foreground	Trait-specific		This work	Indel
MSU7_12_10531640_[Indel]	12	10531640	-21	0	Pita2	Foreground	Trait-specific		This work	Indel
MSU7_12_10612782_[Indel]	12	10612782	+>40	0	Pita2	Foreground	Trait-specific	Fingerprinting	This work	Indel
MSU7_12_10697197_[Indel]	12	10697197	-11	0	Pita2	Foreground	Trait-specific		This work	Indel
MSU7_12_10774796_[G/C]	12	10774796	G	C	Pita2	Foreground	Trait-specific		This work	SNP
MSU7_12_10853832_[Indel]	12	10853832	-30	0/-11	Pita2	Foreground	Trait-specific		This work	Indel
MSU7_12_11069613_[Indel]	12	11069613	-11	0	Pita2	Foreground	Trait-specific		This work	Indel
MSU6_1_34198604_[Indel]	1	34199640	-11	0	qNa1L	Background-recomb	Trait-specific		This work	Indel
MSU6_Chr01_37175070	1	37176113	T	G	qNa1L	Background-recomb	Trait-specific		This work	SNP
MSU6_1_37237133_[Indel]	1	37238176	0	-13	qNa1L	Background-recomb	Trait-specific		This work	Indel
JDP_Chr01_37332506	1	37333549	C	T	qNa1L	Background-recomb	Trait-specific		This work	SNP
JDP_Chr01_37335900	1	37336943	G	T	qNa1L	Background-recomb	Trait-specific		This work	SNP
MSU6_Chr01_37363350	1	37364393	C	G	qNa1L	Background-recomb	Trait-specific		This work	SNP
MSU6_1_37415864_[Indel]	1	37416907	-43	0	qNa1L	Background-recomb	Trait-specific		This work	Indel
MSU6_1_37541965_[Indel]	1	37543008	-12	0	qNa1L	Background-recomb	Trait-specific		This work	Indel
MSU6_1_37579406_[Indel]	1	37580449	+7	0	qNa1L	Background-recomb	Trait-specific		This work	Indel
MSU6_1_37588170_[Indel]	1	37589213	0	+12	qNa1L	Background-recomb	Trait-specific		This work	Indel
MSU6_1_37698557_[Indel]	1	37699597	0	-16	qNa1L	Background-recomb	Trait-specific		This work	Indel
MSU6_1_37724349_[Indel]	1	37725392	-7	0	qNa1L	Background-recomb	Trait-specific		This work	Indel
MSU6_1_37781439_[G/A]	1	37782482	A	G	qNa1L	Foreground	Trait-specific	Fingerprinting	This work	SNP
MSU6_1_37796891_[Indel]	1	37797934	-12	0	qNa1L	Foreground	Trait-specific	Fingerprinting	This work	Indel
MSU6_Chr01_37804554	1	37805597	C	A	qNa1L	Foreground	Trait-specific		This work	SNP
MSU6_1_37903562_[Indel]	1	37904605	GATAAAGA	G	qNa1L	Foreground	Trait-specific		This work	Indel

Supplemental Table 4. List of marker positions interrogated for assessing biological accuracy and breeding metrics. All positions are relative to the MSU7 reference genome

Marker name	Chro-mosome	Position	Favourable allele	Unfavourable allele	Target QTL	Marker linkage	Specificity	Fingerprinting?	Source	Marker type
MSU6_1_37918160_[Indel]	1	37919203	0	-14	qNa1L	Foreground	Trait-specific		This work	Indel
MSU7_1_39300173_[Indel]	1	39300173	-17	0	qNa1L	Foreground	Trait-specific		This work	Indel
MSU6_Chr01_39312420	1	39313463	A	G	qNa1L	Foreground	Trait-specific		This work	SNP
MSU7_1_39400936_[Indel]	1	39400936	0	-21	qNa1L	Foreground	Trait-specific		This work	Indel
MSU7_1_39500179_[Indel]	1	39500179	0	-8	qNa1L	Foreground	Trait-specific		This work	Indel
1_39501901	1	39502944	-32	0	qNa1L	Foreground	Trait-specific		This work	Indel
MSU7_1_39708933_[Indel]	1	39708933	-7	0	qNa1L	Foreground	Trait-specific		This work	Indel
MSU6_Chr01_39795221	1	39796264	G	A	qNa1L	Foreground	Trait-specific		This work	SNP
MSU6_1_39825482_[T/C]	1	39826525	C	T	qNa1L	Foreground	Trait-specific	Fingerprinting	This work	SNP
MSU7_1_39875419_[Indel]	1	39875419	0	-12	qNa1L	Foreground	Trait-specific		This work	Indel
MSU6_Chr01_39886164	1	39887207	A	G	qNa1L	Foreground	Trait-specific		This work	SNP
MSU7_1_39907622_[Indel]	1	39907622	-5	0	qNa1L	Foreground	Trait-specific		This work	Indel
MSU7_1_39975106_[Indel]	1	39975106	0	-36	qNa1L	Foreground	Trait-specific		This work	Indel
MSU7_1_40322404_[Indel]	1	40322404	-11	0	qNa1L	Foreground	Trait-specific		This work	Indel
MSU6_1_40361915_[G/A]	1	40362958	A	G	qNa1L	Foreground	Trait-specific	Fingerprinting	This work	SNP
MSU7_1_40371714_[Indel]	1	40371714	-20	0	qNa1L	Foreground	Trait-specific	Fingerprinting	This work	Indel
MSU7_1_40406873_[T/C]	1	40406873	T	C	qNa1L	Foreground	Trait-specific	Fingerprinting	This work	SNP
MSU7_1_40410995_[Indel]	1	40410995	+13	0	qNa1L	Foreground	Trait-specific	Fingerprinting	This work	Indel
MSU7_1_40426459_[Indel]	1	40426459	-13	0	qNa1L	Foreground	Trait-specific	Fingerprinting	This work	Indel
MSU7_1_40477292_[-5/0]	1	40477292	-5	0	qNa1L	Foreground	Trait-specific	Fingerprinting	This work	Indel
MSU7_1_40497261_[Indel]	1	40497261	-124	0	qNa1L	Foreground	Trait-specific		This work	Indel
MSU7_1_40611906_[T/A]	1	40611906	T	A	qNa1L	Foreground	Trait-specific	Fingerprinting	This work	SNP
MSU7_1_40614286_[Indel]	1	40614286	-11	0	qNa1L	Foreground	Trait-specific	Fingerprinting	This work	Indel
MSU6_Chr01_42018165	1	42019208	T	C	qNa1L	Background-recomb	Trait-specific		This work	SNP
MSU6_Chr01_42240347	1	42241391	A	G	qNa1L	Background-recomb	Trait-specific		This work	SNP
MSU6_Chr01_42877064	1	42878108	T	A	qNa1L	Background-recomb	Trait-specific		This work	SNP
MSU6_Chr01_43185541	1	43186585	T	G	qNa1L	Background-recomb	Trait-specific		This work	SNP
MSU7_1_39084567_[Indel]	1	39084567	-14	0	qSCT1	Foreground	Trait-specific	Fingerprinting	This work	Indel
MSU7_1_39188650_[C/T]	1	39188650	C	T	qSCT1	Foreground	Trait-specific	Fingerprinting	This work	SNP
MSU7_1_39190098_[Indel]	1	39190098	0	-9	qSCT1	Foreground	Trait-specific	Fingerprinting	This work	Indel

Supplemental Table 4. List of marker positions interrogated for assessing biological accuracy and breeding metrics. All positions are relative to the MSU7 reference genome

Marker name	Chro-mosome	Position	Favourable allele	Unfavourable allele	Target QTL	Marker linkage	Specificity	Fingerprinting?	Source	Marker type
MSU7_1_39213471_[A/C]	1	39213471	A	C	qSCT1	Foreground	Trait-specific	Fingerprinting	This work	SNP
MSU7_1_39246500_[A/T]	1	39246500	A	T	qSCT1	Foreground	Trait-specific	Fingerprinting	This work	SNP
MSU7_1_39547975_[Indel]	1	39547975	-19	0	qSCT1	Foreground	Trait-specific	Fingerprinting	This work	Indel
MSU7_1_39553459_[T/G]	1	39553459	T	G	qSCT1	Foreground	Trait-specific	Fingerprinting	This work	SNP
MSU7_1_39553546_[C/T]	1	39553546	C	T	qSCT1	Foreground	Trait-specific	Fingerprinting	This work	SNP
MSU7_1_39554641_[Indel]	1	39554641	-16	0	qSCT1	Foreground	Trait-specific	Fingerprinting	This work	Indel
MSU7_1_39580791_[Indel]	1	39580791	0	+5	qSCT1	Foreground	Trait-specific	Fingerprinting	This work	Indel
MSU7_1_11235353_[Indel]	1	11235353	-11	0	Saltol	Foreground	Trait-specific		This work	Indel
MSU7_1_11434110_[Indel]	1	11434110	-9	0	Saltol	Foreground	Trait-specific		This work	Indel
jdpSKC1.5	1	11460344	T	G	Saltol	Foreground	Trait-specific		This work	SNP
MSU7_1_11460918_[Indel]	1	11460918	-14	0	Saltol	Foreground	Trait-specific		This work	Indel
jdpSKC1.9	1	11462124	T	C	Saltol	Foreground	Trait-specific		This work	SNP
MSU7_1_11462282_[C/G]	1	11462282	C	G	Saltol	Foreground	Trait-specific		This work	SNP
jdpSKC1.4	1	11462725	T	C	Saltol	Foreground	Trait-specific	Fingerprinting	This work	SNP
jdpSKC1.3	1	11463299	A	T	Saltol	Foreground	Trait-specific		This work	SNP
jdpSKC1.2	1	11463595	A	C	Saltol	Foreground	Trait-specific		This work	SNP
MSU7_1_11469391_[Indel]	1	11469391	-36	0	Saltol	Foreground	Trait-specific		This work	Indel
MSU7_1_11583669_[Indel]	1	11583669	-29	0	Saltol	Foreground	Trait-specific		This work	Indel
MSU7_6_27480424_[-9/0]	6	27480424	-9	0	SCM2	Foreground	Trait-specific	Fingerprinting	This work	Indel
MSU7_6_27480778_[G/C]	6	27480778	C	G	SCM2	Foreground	Trait-specific	Fingerprinting	This work	SNP
MSU7_6_27481548_[C/T]	6	27481548	T	C	SCM2	Foreground	Trait-specific	Fingerprinting	This work	SNP
MSU7_6_27481897_[0/+12]	6	27481897	T	TATTTTTATT	SCM2	Foreground	Trait-specific		This work	SNP
MSU7_9_6248200_[T/C]	9	6248200	T	C	Sub1	Foreground	Trait-specific	Fingerprinting	This work	SNP
MSU7_9_6371914_[Indel]	9	6371914	-7	0	Sub1	Foreground	Trait-specific	Fingerprinting	This work	Indel
MSU7_9_6374718_[A/G]	9	6374718	A	G	Sub1	Foreground	Trait-specific	Fingerprinting	This work	SNP
MSU7_9_6381979_[A/G]	9	6381979	A	G	Sub1	Foreground	Trait-specific	Fingerprinting	This work	SNP
MSU7_9_6605992_[Indel]	9	6605992	-8	0	Sub1	Foreground	Trait-specific	Fingerprinting	This work	Indel
MSU7_12_16368123_[G/T]	12	16368123	G	T	SWEET13	Background-recomb	Trait-specific		This work	SNP
MSU7_12_16575958_[Indel]	12	16575958	0	-10	SWEET13	Background-recomb	Trait-specific		This work	Indel
MSU7_12_17181133_[T/C]	12	17181133	T	C	SWEET13	Background-recomb	Trait-specific		This work	SNP

Supplemental Table 4. List of marker positions interrogated for assessing biological accuracy and breeding metrics. All positions are relative to the MSU7 reference genome

Marker name	Chro-mosome	Position	Favourable allele	Unfavourable allele	Target QTL	Marker linkage	Specificity	Fingerprinting?	Source	Marker type
MSU7_12_17304011_[Indel]	12	17304011	-10	0	SWEET13	Foreground	Trait-specific		This work	Indel
MSU7_12_17304146_[C/A]	12	17304146	C	A	SWEET13	Foreground	Trait-specific		This work	SNP
MSU7_12_17306920_[A/C]	12	17306920	A	C	SWEET13	Foreground	Trait-specific		This work	SNP
MSU7_12_17487095_[Indel]	12	17487095	0	-32	SWEET13	Background-recomb	Trait-specific		This work	Indel
MSU7_12_17494145_[C/A]	12	17494145	C	A	SWEET13	Background-recomb	Trait-specific		This work	SNP
MSU7_11_17906805_[Indel]	11	17906805	0	-10	SWEET14	Background-recomb	Trait-specific		This work	Indel
MSU7_11_17920870_[T/A]	11	17920870	T	A	SWEET14	Background-recomb	Trait-specific		This work	SNP
MSU7_11_17939282_[C/T]	11	17939282	C	T	SWEET14	Background-recomb	Trait-specific		This work	SNP
MSU7_11_18173234_[Indel]	11	18173234	0	-11	SWEET14	Foreground	Trait-specific		This work	Indel
MSU7_11_18179253_[A/G]	11	18179253	A	G	SWEET14	Foreground	Trait-specific		This work	SNP
MSU7_11_18203671_[Indel]	11	18203671	0	-10	SWEET14	Foreground	Trait-specific		This work	Indel
MSU7_11_18244383_[Indel]	11	18244383	-17	0	SWEET14	Foreground	Trait-specific		This work	Indel
MSU7_11_18463107_[C/T]	11	18463107	C	T	SWEET14	Background-recomb	Trait-specific		This work	SNP
MSU7_11_18490050_[A/T]	11	18490050	A	T	SWEET14	Background-recomb	Trait-specific		This work	SNP
MSU7_6_25092328_[Indel]	6	25092328	-7	0	TGW6	Foreground	Trait-specific		This work	Indel
MSU6_6_25091603_[C/T]	6	25092600	T	C	TGW6	Foreground	Trait-specific		This work	SNP
MSU7_6_25093553_[Indel]	6	25093553	-1	0	TGW6	Foreground	Trait-specific	Fingerprinting	This work	Indel
MSU6_6_25093447_[A/G]	6	25094444	G	A	TGW6	Foreground	Trait-specific		This work	SNP
MSU7_7_22006430_[T/C]	7	22006430	T	C	TSV1	Foreground	Trait-specific		This work	SNP
MSU7_7_22041970_[T/G]	7	22041970	T	G	TSV1	Foreground	Trait-specific		This work	SNP
MSU7_7_22098691_[Indel]	7	22098691	0	-7	TSV1	Foreground	Trait-specific		This work	Indel
MSU7_7_22102272_[Indel]	7	22102272	-9	0	TSV1	Foreground	Trait-specific		This work	Indel
MSU7_7_22117064_[A/G]	7	22117064	A	G	TSV1	Foreground	Trait-specific		This work	SNP
MSU6_7_22117466[T/C]	7	22118459	C	T	TSV1	Foreground	Trait-specific	Fingerprinting	This work	SNP
MSU6_7_22118345[G/T]	7	22119338	T	G	TSV1	Foreground	Trait-specific		This work	SNP
MSU7_7_22119346_[A/T]	7	22119346	A	T	TSV1	Foreground	Trait-specific		This work	SNP
MSU7_7_22119347_[A/G]	7	22119347	G	A	TSV1	Foreground	Trait-specific		This work	SNP
MSU6_7_22118355[Indel]	7	22119347	-3	0	TSV1	Foreground	Trait-specific		This work	Indel
MSU7_7_22123671_[Indel]	7	22123671	+11	0	TSV1	Foreground	Trait-specific		This work	Indel
MSU7_6_1765761_[T/G]	6	1765761	T	G	Waxy	Foreground	Trait-specific		This work	SNP

Supplemental Table 4. List of marker positions interrogated for assessing biological accuracy and breeding metrics. All positions are relative to the MSU7 reference genome

Marker name	Chro-mosome	Position	Favourable allele	Unfavourable allele	Target QTL	Marker linkage	Specificity	Fingerprinting?	Source	Marker type
MSU7_6_1767284_[Indel]	6	1767284	-16	0	Waxy	Foreground	Trait-specific		This work	Indel
MSU7_6_1768006_[C/A]	6	1768006	C	A	Waxy	Foreground	Trait-specific	Fingerprinting	This work	SNP
MSU7_8_26250376_[Indel]	8	26250376	0	-12	xa13	Background-recomb	Trait-specific		This work	Indel
xa13-SNP3	8	26725734	T	C	xa13	Foreground	Trait-specific		This work	SNP
xa13-SNP2	8	26726585	T	C	xa13	Foreground	Trait-specific		This work	SNP
xa13-SNP1	8	26727222	C	G	xa13	Foreground	Trait-specific		This work	SNP
xa13-pro upstream	8	26729914	-20	0	xa13	Foreground	Trait-specific		This work	Indel
MSU7_8_27519468_[Indel]	8	27519468	0	-12	xa13	Background-recomb	Trait-specific		This work	Indel
MSU7_8_27734755_[Indel]	8	27734755	0	-21	xa13	Background-recomb	Trait-specific		This work	Indel
MSU7_11_19880897_[Indel]	11	19880897	0	-23	Xa21	Background-recomb	Trait-specific		This work	Indel
MSU7_11_19938986_[Indel]	11	19938986	0	-27	Xa21	Background-recomb	Trait-specific		This work	Indel
MSU7_11_19940986_[Indel]	11	19940986	0	-20	Xa21	Background-recomb	Trait-specific		This work	Indel
Xa21-SNP1	11	21273275	G	A	Xa21	Foreground	Trait-specific		This work	SNP
Xa21_indel-1	11	21274459	+29	0	Xa21	Foreground	Trait-specific	Fingerprinting	This work	Indel
Xa21-SNP2	11	21276339	T	C	Xa21	Foreground	Trait-specific		This work	SNP
Xa21-SNP3	11	21276714	C	G	Xa21	Foreground	Trait-specific		This work	SNP
Xa21-SNP4	11	21277004	T	C	Xa21	Foreground	Trait-specific		This work	SNP
MSU7_11_21502281_[T/C]	11	21502281	T	C	Xa21	Background-recomb	Trait-specific		This work	SNP
MSU7_11_22950926_[Indel]	11	22950926	0	-13	Xa21	Background-recomb	Trait-specific		This work	Indel
MSU7_11_22042058_[Indel]	11	22042058	-7	0	Xa23	Foreground	Trait-specific		This work	Indel
MSU7_11_22141523_[A/G]	11	22141523	A	G	Xa23	Foreground	Trait-specific		This work	SNP
MSU7_11_22142546_[Indel]	11	22142546	0	-15	Xa23	Foreground	Trait-specific		This work	Indel
MSU7_11_22175063_[G/T]	11	22175063	G	T	Xa23	Foreground	Trait-specific	Fingerprinting	This work	SNP
MSU7_11_22225387_[T/A]	11	22225387	T	A	Xa23	Foreground	Trait-specific		This work	SNP
MSU7_11_22225882_[G/A]	11	22225882	G	A	Xa23	Foreground	Trait-specific		This work	SNP
MSU7_11_22230977_[Indel]	11	22230977	0	-9	Xa23	Foreground	Trait-specific		This work	Indel
MSU7_11_22261158_[A/T]	11	22261158	A	T	Xa23	Foreground	Trait-specific		This work	SNP
MSU7_11_22309091_[G/A]	11	22309091	G	A	Xa23	Foreground	Trait-specific		This work	SNP
MSU7_11_26406261_[Indel]	11	26406261	-32	0	Xa4	Background-recomb	Trait-specific		This work	Indel
MSU7_11_27119887_[Indel]	11	27119887	-15	0	Xa4	Background-recomb	Trait-specific	Fingerprinting	This work	Indel

Supplemental Table 4. List of marker positions interrogated for assessing biological accuracy and breeding metrics. All positions are relative to the MSU7 reference genome

Marker name	Chro-mosome	Position	Favourable allele	Unfavourable allele	Target QTL	Marker linkage	Specificity	Fingerprinting?	Source	Marker type
MSU7_11_27122941_[T/A]	11	27122941	T	A	Xa4	Foreground	Trait-specific	Fingerprinting	This work	SNP
MSU7_11_27265090_[Indel]	11	27265090	-43	0	Xa4	Background-recomb	Trait-specific	Fingerprinting	This work	Indel
MSU7_11_27353895_[Indel]	11	27353895	-16	0	Xa4	Background-recomb	Trait-specific	Fingerprinting	This work	Indel
MSU7_11_27585379_[Indel]	11	27585379	-10	0	Xa4	Foreground	Trait-specific	Fingerprinting	This work	Indel
MSU7_11_28005031_[Indel]	11	28005031	-13	0	Xa4	Foreground	Trait-specific		This work	Indel
MSU7_11_28009751_[T/C]	11	28009751	T	C	Xa4	Foreground	Trait-specific		This work	SNP
MSU7_11_28124705_[C/G]	11	28124705	C	G	Xa4	Foreground	Trait-specific		This work	SNP
MSU7_11_28224947_[Indel]	11	28224947	-15	0	Xa4	Foreground	Trait-specific		This work	Indel
MSU7_11_28365515_[Indel]	11	28365515	-9	0	Xa4	Foreground	Trait-specific		This work	Indel
MSU7_11_28704769_[T/G]	11	28704769	T	G	Xa4	Foreground	Trait-specific	Fingerprinting	This work	SNP
MSU7_5_436945_[A/G]	5	436945	A	G	Xa5	Foreground	Trait-specific		This work	SNP
MSU7_5_438578_[T/C]	5	438578	T	C	Xa5	Foreground	Trait-specific		This work	SNP
MSU7_5_438902_[0/-35]	5	438902	0	-35	Xa5	Foreground	Trait-specific		This work	Indel
MSU7_6_27891614_[C/G]	6	27891614	C	G	Xa7	Foreground	Trait-specific		This work	SNP
MSU7_6_27891667_[Indel]	6	27891667	-14	0	Xa7	Foreground	Trait-specific	Fingerprinting	This work	Indel
MSU7_6_27912418_[Indel]	6	27912418	-7	0	Xa7	Foreground	Trait-specific	Fingerprinting	This work	Indel
MSU7_6_27917038_[Indel]	6	27917038	-16	0	Xa7	Foreground	Trait-specific	Fingerprinting	This work	Indel
MSU7_6_27919547_[T/G]	6	27919547	T	G	Xa7	Foreground	Trait-specific	Fingerprinting	This work	SNP
MSU7_6_27959630_[Indel]	6	27959630	-8	0	Xa7	Foreground	Trait-specific	Fingerprinting	This work	Indel
MSU7_6_28010904_[Indel]	6	28010904	-8	0	Xa7	Foreground	Trait-specific	Fingerprinting	This work	Indel
MSU7_6_28011120_[G/A]	6	28011120	G	A	Xa7	Foreground	Trait-specific	Fingerprinting	This work	SNP
MSU7_6_28016299_[Indel]	6	28016299	-19	0	Xa7	Foreground	Trait-specific	Fingerprinting	This work	Indel
MSU7_6_28082926_[Indel]	6	28082926	0	-11	Xa7	Foreground	Trait-specific		This work	Indel
MSU7_6_28187269_[A/G]	6	28187269	A	G	Xa7	Foreground	Trait-specific	Fingerprinting	This work	SNP
