# Supplementary material for: Criteria for evaluating molecular markers: Comprehensive quality metrics to improve marker-assisted selection
Source: PLoS One. 2019 Jan 15;14(1):e0210529. doi: 10.1371/journal.pone.0210529 (PMC6333336; doi:10.1371/journal.pone.0210529)
Supplement: S5 Table — (PDF) [file pone.0210529.s005.pdf]

Supplemental Table 5. List of QTL examined, with start and end positions.

Trait category	Target QTL	Chromosome	Start	End
Abiotic stress	AG1	9	12036453	12545658
Abiotic stress	COLD1	4	30174083	31052578
Abiotic stress	LTG1	2	24693023	24787561
Abiotic stress	qSCT1	1	39076741	40477292
Abiotic stress	DTY1.1	1	37925965	40473982
Abiotic stress	DTY12.1	12	17066946	17571574
Abiotic stress	DTY2.1	2	9570228	12035419
Abiotic stress	DTY2.2	2	209765	8882316
Abiotic stress	DTY3.1	3	28587948	31393778
Abiotic stress	DTY3.2	3	180286	13032396
Abiotic stress	DTY4.1	4	59946	3557787
Abiotic stress	HTSF4.1	4	17474229	20149751
Abiotic stress	qNa1L	1	37782482	40997165
Abiotic stress	Saltol	1	11110274	11739003
Abiotic stress	Sub1	9	6191930	6774928
Biotic stress	Pi35(t)	1	32898485	33321905
Biotic stress	Pi54	11	24843208	25974491
Biotic stress	Pi9	6	10049864	10677685
Biotic stress	Pita2	12	9177624	11069613
Biotic stress	xa13	8	26316287	27300242
Biotic stress	Xa21	11	19941316	21508416
Biotic stress	Xa23	11	22008834	22923998
Biotic stress	Xa4	11	25974491	28804914
Biotic stress	Xa5	5	255323	767585
Biotic stress	Xa7	6	27609767	28187269
Biotic stress	BPH17	4	6835634	7195884
Biotic stress	BPH3	6	1272777	1768006
Biotic stress	TSV1	7	21998892	22381105
Grain quality	frg-1	8	20213731	20413803
Grain quality	SSIIa (gelatinisation temperature)	6	6508525	6891960
Grain quality	GW5/SW5	5	5256714	5588965
Grain quality	Waxy	6	1501961	1934659
Grain quality	Chalk5	5	3253516	3470087
Grain quality	PGWC8-2	8	25757847	26074700
Grain quality	GS3	3	16688962	16889939
Maturity	DTH8	8	4101332	4603854
Maturity	Hd9	3	996213	1851409
Yield components	DEP1	8	16394577	16523573
Yield components	Gn1a	1	4949689	5608892
Yield components	NAL1	4	30307709	31419137
Yield components	SCM2	6	27371995	27609767
Yield components	TGW6	6	24875490	25387111
