# Supplementary material for: Criteria for evaluating molecular markers: Comprehensive quality metrics to improve marker-assisted selection
Source: PLoS One. 2019 Jan 15;14(1):e0210529. doi: 10.1371/journal.pone.0210529 (PMC6333336; doi:10.1371/journal.pone.0210529)
Supplement: S1 File — A more detailed explanation of the theory behind Figs 2 and 3. (PDF) [file pone.0210529.s006.pdf]

Supplemental Text

Evolution of QTL and markers

During the evolutionary history of a species, mutations arise throughout the genome, resulting in new allelic variants of a gene. Each allele has one or more mutations that accumulate over time and distinguish it from its ancestral form and other extant alleles. These polymorphisms are often used to inform the construction of a phylogenetic trees, revealing the relative divergence times between the observed alleles. If at some point a mutation arises that alters the phenotype of an individual for a specific trait, a genetic difference for the trait arises and a quantitative trait locus is born. This mutation will thus represent a divergence within the allelic lineage of a gene, producing a *derived allele* with a status as either favourable or unfavourable. Any polymorphism in or around the causative gene – whether previously accumulated or subsequently derived – are tightly linked to the causal gene and become potential markers for the new QTL. However not all will have the same level of association; mutations that pre-date the emergence of the QTL allele will already exist at some frequency in the gene pool and as such may not be in high linkage disequilibrium with the new QTL allele despite being tightly linked. If these pre-existing polymorphisms are used as markers for the QTL, they will classify some individuals as QTL[+] when it is in fact absent – i.e. they will give false-positive results. Likewise mutations that post-date the emergence of the QTL will have a lower frequency in the gene pool than the causative mutation. If such variants are used as markers, they may score an individual as QTL[-], despite having a favourable (QTL[+]) allele at the causal locus, i.e. false-negative results. Over time, as selection, drift, non-random mating, and other genetic forces act on the new QTL allele, the allele frequency will change in the gene pool as well. Therefore the challenge of marker design is to identify which polymorphisms will correctly classify as many of these alleles as donor or recipient as possible.

In an ideal situation, QTL mapping in multiple populations (or association mapping in a panel of diverse lines) results in knowledge of multiple donor and recipient lines, each potentially possessing different alleles of a given gene. This enables at least some alleles to be reliably characterised as representing the favourable or unfavourable states, and resulting in a situation like that presented in Figure 2. This makes it possible to hypothesise which polymorphisms will reliably categorise favourable and unfavourable alleles. In the above example polymorphism number 6, 19, 20, and 21 will reliably distinguish the known donor allele from all the known recipients; which particular polymorphism is best among them depends on whether allele F is determined to be a favourable or an unfavourable allele. A more general illustration involving several possible evolutionary scenarios is given in Figure 3, along with the mutations that would make the most reliable markers. In each case the causal mutation gives rise to an allele that has gained either favourable or unfavourable status. Note that the causal mutation could give rise to *either* a favourable or an unfavourable allele. For example a mutation may disrupt the function of an important gene, resulting in a loss of activity and an unfavourable phenotype.

However the causal mutation is always a derived allele – descended from one of the previously-existing alleles of the gene, and distinguished by the causal mutation.
